# Latent profile analysis of eHealth literacy and its sociodemographic correlates: a cross-sectional study

**DOI:** 10.3389/fpubh.2026.1904244

**Published:** 2026-07-27

**Authors:** Hongmin Li, Min Jiao, Dongxu Li

**Affiliations:** School of Public Health, Jining Medical University, Jining, China

**Keywords:** cross-sectional study, eHealth literacy heterogeneity, eHealth literacy scale(eHEALS), latent profile analysis, sociodemographic correlates

## Abstract

**Objective:**

This study used Latent Profile Analysis (LPA) to classify Chinese adults with access to digital health information into distinct eHealth literacy subgroups and depict their multidimensional ability traits. We further examined cross-sectional sociodemographic and health-related correlates of subgroup affiliation, generating descriptive data that may serve as a preliminary reference for future stratified digital health outreach research.

**Methods:**

A cross-sectional online survey was conducted among Chinese residents between June and July 2023. The widely used Chinese eHealth literacy scale (eHEALS) measured three dimensions of digital health literacy via 1–5 Likert items. Optimal latent class number was determined using AIC, BIC, entropy and BLRT. Weighted multinomial logistic regression, adjusted by LPA posterior probabilities, was adopted to explore associated factors. LPA was performed in R 4.2.1, and regression analysis in Stata 17.0.

**Results:**

In total, 1,391 valid questionnaires were collected for analysis. The five-class model was selected as optimal, identifying five eHealth literacy latent profiles: Low eHealth Literacy (11.29%), Moderate-Low Balanced eHealth Literacy (18.26%), Moderate-High Balanced eHealth Literacy (30.91%), High Balanced eHealth Literacy (29.69%), and Application-Preferred Moderate-High eHealth Literacy (9.85%). Regression results revealed subgroup-specific correlational patterns. Higher educational attainment was positively correlated with membership in the Application-Preferred Moderate-High eHealth Literacy profile (OR range: 3.774–4.552, all *p* < 0.01), while advancing age was negatively associated with membership in this profile (OR = 0.936, *p* < 0.001). Participants with chronic diseases or commercial medical insurance were more likely to be categorized into the Application-Preferred Moderate-High eHealth Literacy subgroup (OR = 3.408, *p* < 0.001). Urban residence was negatively associated with the Application-Preferred Moderate-High profile (OR = 0.579, *p* = 0.044), while non-employed status was negatively associated with the Moderate-High Balanced profile (OR = 0.535, *p* = 0.007).

**Conclusion:**

Different eHealth literacy latent profiles exhibited unique cross-sectional correlational patterns with multiple sociodemographic and health characteristics among digitally accessible Chinese adults. Educational attainment consistently showed positive correlational links with higher self-reported digital health capacity, particularly for the Application-Preferred Moderate-High profile, while age and non-employment showed divergent correlations across subgroups. Household income demonstrated a profile-specific correlational pattern: lower household income was only associated with increased odds of belonging to the Application-Preferred Moderate-High subgroup versus the Low eHealth Literacy reference group.

## 1 Introduction

With the arrival of the digital health era, the Internet and mobile technologies have become core channels for the public to access health information, manage health conditions, and make health decisions (1). Electronic health literacy (eHealth literacy) refers to the capacity to seek, comprehend, evaluate, and apply health information from online sources to address health-related issues (2). Individuals with higher eHealth literacy levels tend to exhibit better health self-management, active engagement in medical decision-making, and favorable mental status and quality of life (3). As an important cross-sectional correlate of health, eHealth literacy is closely associated with improved health-related behaviours, medication adherence, and overall health outcomes (4–8).

Numerous tools have been developed to assess eHealth literacy (9), among which the eHealth Literacy Scale (eHEALS) is the most widely used instrument (10, 11). As a multidimensional construct, eHEALS is commonly used to evaluate overall eHealth literacy levels, but most existing studies treat eHealth literacy as a homogeneous construct, analyzing it through total scores or dimension means. This approach overlooks the potential heterogeneity among individuals, ignoring the possibility of uneven development in different dimensions of eHealth literacy (e.g., proficient application but poor critical evaluation ability).

Latent Profile Analysis (LPA), a person-centered statistical method, can identify homogeneous subgroups (profiles) within a heterogeneous population based on multiple observable variables (12). It shifts the research perspective from relationships between variables to overall patterns of individuals, making it suitable for exploring the latent heterogeneity of eHealth literacy. LPA has been widely applied in fields such as mental health (13–16), smartphone use behaviour (17, 18), and digital literacy (19–21), but its application in eHealth literacy research remains insufficient, especially regarding the identification of eHealth literacy profiles and their associated sociodemographic and health-related correlates.

A systematic review has shown that personalized interventions can significantly improve eHealth literacy levels (22), however, implementing such interventions requires clear identification of heterogeneous eHealth literacy subgroups and their associated sociodemographic and health-related correlates. Accordingly, this cross-sectional study aimed to:

(1) Identify latent eHealth literacy profiles via LPA;
(2) Examine associations between demographic, socioeconomic, and health-related variables and latent profile membership, to provide observational empirical evidence for developing stratified digital health promotion strategies and advancing health equity.

## 2 Methods

### 2.1 Study design and setting

We conducted a cross-sectional study among Chinese online adult participants using an online self-administered questionnaire. Data were collected via Wenjuanxing^1^ from June to July 2023. Using convenience sampling, we recruited respondents aged 18 years or older who were able to access and retrieve digital health information via health-related mobile applications or online health platforms. Individuals without basic digital access or the capacity to obtain online health information were not eligible for this study.

**Figure 1:**
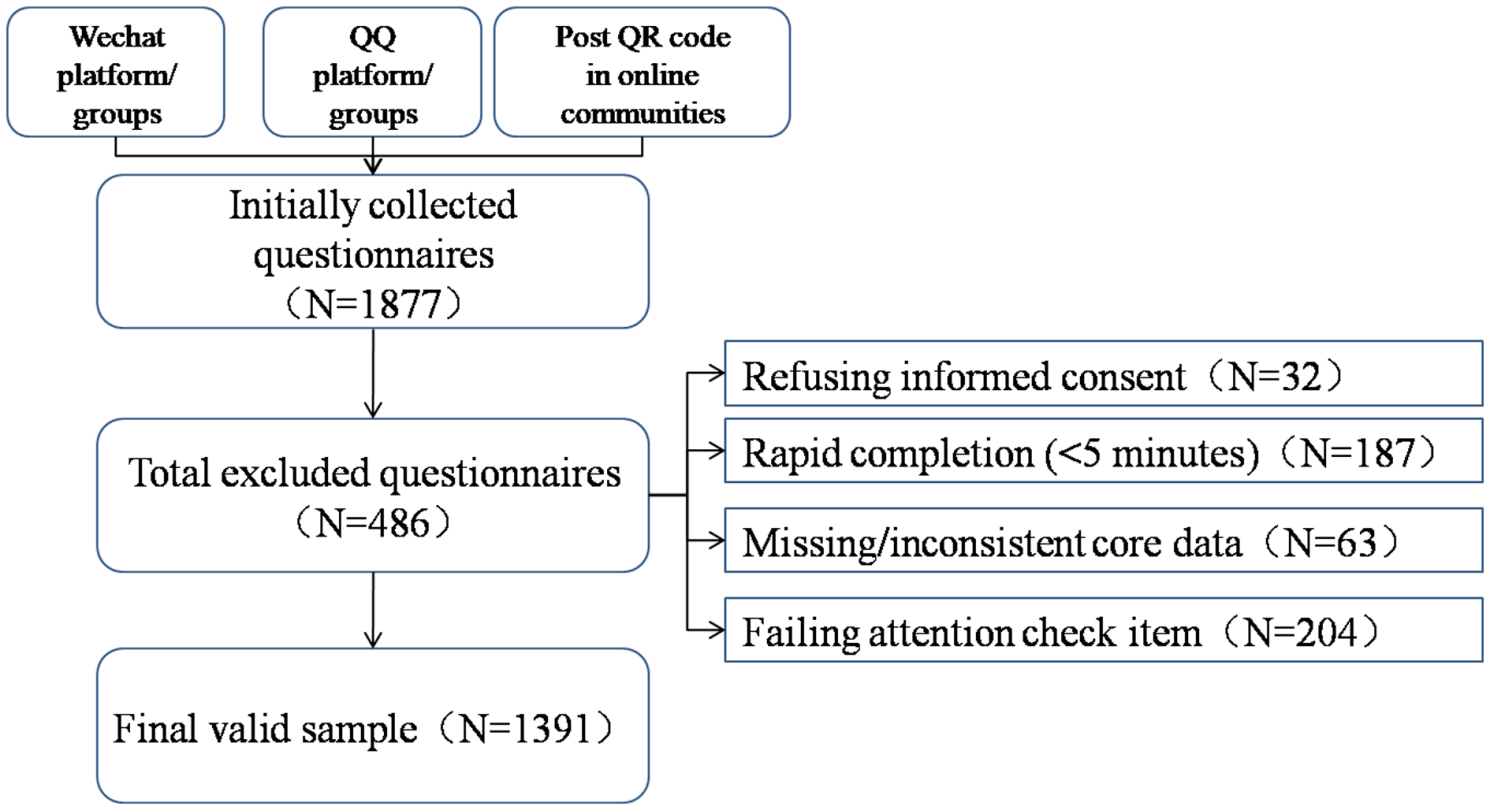
Flowchart of participant recruitment and sample screening procedure.

Participants were excluded if they: (1) declined to provide informed consent; (2) finished the questionnaire within fewer than 5 min, which indicated careless random responding; (3) submitted responses with inconsistent or missing core data, such as incomplete eHEALS dimension scores or unreported demographic information; (4) failed the embedded attention-check item (instructing respondents to select the option “strongly agree”) designed to screen out careless and invalid submissions. Participant recruitment and sample screening procedures are presented in Figure 1.

We adopted convenience and snowball sampling to distribute the online questionnaire link through WeChat groups, WeChat Moments, QQ groups, and QR codes published in online community platforms. The Wenjuanxing survey platform enabled IP address restriction to prevent repeated submissions from the same device or network. No financial or material incentives were provided to participants, and all respondents gave voluntary informed consent prior to completing the questionnaire. Initially, 1,877 questionnaires were retrieved. In total, 486 responses were excluded for predefined reasons: 32 responses from participants who refused to provide informed consent; 187 questionnaires completed in fewer than 5 min, indicating careless random responding; 63 submissions with missing or inconsistent core demographic and eHEALS scale data; 204 questionnaires that failed the embedded attention-check item, representing the leading cause of sample exclusion. The remaining 1,391 valid questionnaires were included for latent profile analysis and weighted multinomial logistic regression.

It should be noted that our sample only represents Chinese adults with accessible digital health resources, and study findings cannot be directly generalized to all Chinese residents, particularly populations lacking internet access or basic digital operational skills.

### 2.2 Measurements

#### 2.2.1 Primary outcome: electronic health literacy

Electronic health literacy was assessed using the Chinese version of the eHEALS, which has been adapted for the Chinese population with good reliability and validity (Cronbach’s *α* = 0.913) (23). The eHEALS consists of 8 items, divided into three dimensions: (1) Application Ability (items 1–5), items capture respondents’ capacity to search for and utilize online health information; (2) Critical Evaluation Ability (items 6–7), items measure respondents’ capacity to assess the credibility of digital health content; (3) Decision-Making Ability (item 8), items gauge respondents’ capacity to utilize online health information for personal health judgments. Each item is scored on a 5-point Likert scale (1 = strongly disagree, 5 = strongly agree). Comprehensive psychometric analyzes were conducted in the present sample (*N* = 1,391) to evaluate the measurement properties of the scale. Internal consistency was examined using both Cronbach’s *α* and McDonald’s *ω*; the omega coefficient does not require the tau-equivalence assumption that Cronbach’s α relies on. The full 8-item scale yielded Cronbach’s α = 0.9507 and McDonald’s ω = 0.9509, with detailed reliability results presented in Supplementary Table S1.

Principal component exploratory factor analysis (EFA) was conducted on all eight items. Only one factor with an eigenvalue greater than 1 was extracted, explaining 74.67% of total variance. All standardised item loadings ranged from 0.816 to 0.907, showing high convergent indicator correlations and consistent with the original unidimensional theoretical structure of the eHEALS scale (Supplementary Tables S2, S3). We further performed confirmatory factor analysis (CFA) to compare the unidimensional 8-item model and two-factor 7-item model (application + critical evaluation). The two-factor model demonstrated substantially better fit indices [(χ^2^ (13) = 253.25), CFI = 0.974, TLI = 0.958, RMSEA = 0.115, SRMR = 0.024] relative to the unidimensional structure [(χ^2^ (20) = 854.24), CFI = 0.923, TLI = 0.892, RMSEA = 0.173, SRMR = 0.043]. Nevertheless, the two-factor model yielded an RMSEA value of 0.115, exceeding commonly accepted cutoffs for adequate model fit. Although all standardised factor loadings were above 0.7 (*p* < 0.001), we refrain from overinterpreting these outputs as supportive evidence of sound discriminant validity in the current sample (Supplementary Table S4).

Notably, the Critical Evaluation subdimension includes only two items, and the Decision-Making dimension is defined by a single item. This sparse distribution of items restricts the possibility of deriving a robust three-factor measurement structure, and the current sample does not provide sufficient psychometric evidence to support a three-dimensional factor solution. For this reason, the three-dimensional grouping adopted in the current study represents a theoretically driven analytical classification tailored for LAP, rather than a three-factor measurement structure derived from sample-specific psychometric evaluation. We computed the mean score for each theoretically predefined dimension for all subsequent statistical analyzes, where higher average scores represent a higher level of corresponding electronic health literacy.

#### 2.2.2 Independent variables and control variables

Demographic, socioeconomic, and health-related variables were included as covariates in the analysis, covering the following indicators: gender, age, residential location (urban vs. rural), educational level (primary school and below, junior high school, senior high school, college degree and above), employment status (employed, student, retired, unemployed and other non-working groups), commercial medical insurance status, chronic disease status (defined as a physician-confirmed diagnosis of one or more chronic conditions including hypertension, diabetes, coronary heart disease, and other chronic illnesses). Annual household income was divided into five categories: less than 50,000 CNY, 50,000–99,999 CNY, 100,000–149,999 CNY, 150,000–199,999 CNY, and 200,000 CNY and above.

### 2.3 Statistical analysis

All statistical analyzes adopted a two-stage analytical framework: LPA was conducted in R (version 4.2.1) using the tidyLPA package, whereas weighted multinomial logistic regression and additional sensitivity tests were performed in Stata 17.0. A two-tailed *P* less than 0.05 was considered statistically significant. Frequency and percentage were used to describe categorical variables, and mean ± standard deviation (Mean ± SD) was used to describe continuous variables (e.g., age, eHEALS dimension scores).

LPA was performed to identify distinct latent profiles of eHealth literacy based on the three theoretically derived dimensions of the eHEALS scale. We employed constrained Model 1 (equal variances and zero cross-class covariances). This specification is commonly adopted for Likert-type data, as it may help reduce estimation bias and the risk of model overfitting. To assess the stability of the solution, we repeated the analysis with three independent random seeds (123, 456, and 789), all of which converged to the identical 5-class solution. The optimal number of profiles was determined by multiple fit indices: (1) Bayesian Information Criterion (BIC), Akaike Information Criterion (AIC) (lower values indicate better model fit); (2) Entropy (values closer to 1 indicate higher classification accuracy, Entropy ≥0.8 is considered acceptable); (3) Average posterior probability per latent class, where a minimum value of ≥0.7 serves as the benchmark for satisfactory classification reliability; (4) Bootstrap Likelihood Ratio Test (BLRT), where a *p*-value < 0.05 indicates that the k-class model fits the data significantly better than the (k − 1)-class alternative; (5) Substantive interpretability: valid latent profiles were required to have distinct interpretative features and account for no less than 5% of the total analytical sample.

To address the methodological concern that the five-profile solution might be artificially driven by the single-item decision-making dimension, we conducted two targeted sensitivity analyzes. First, item-level LPA was performed using all eight raw eHEALS items. The five-class solution remained optimal with high entropy of 0.988, and the subgroup structure was highly consistent with the dimension-based primary analysis. Second, we re-estimated LPA after excluding the decision-making dimension. Although the six-class model showed the lowest BIC value, it exhibited severe overfitting with the smallest subgroup constituting only 1.58% of participants and was therefore discarded (Supplementary Tables S5).

The five-class solution presented clear hierarchical gradients of eHealth literacy, providing empirical support for this classification structure. Importantly, despite consistent results across sensitivity tests, the primary dimension-based LPA solution exhibited zero within-profile standard deviation for the single-item decision-making indicator across several latent classes. This suggests that the five-profile structure, though statistically supported by multiple fit indices and sensitivity analyzes, may still partially reflect the discrete, single-item nature of the decision-making measure. We do not rule out residual measurement influence from this indicator, and this concern cannot be considered fully resolved by the current sensitivity analyzes.

For the final five-profile model, the overall entropy was 0.952, and the diagonal average posterior probabilities for correct classification were 0.538, 0.882, 0.902, 0.909, and 0.952 for the five profiles, with a range of 0.538–0.952, indicating good overall classification certainty. Notably, Class 1 (Low eHealth Literacy) exhibited a diagonal probability of 0.538, which is below the conventional 0.70 threshold, indicating that this profile has relatively lower classification certainty compared with the other four profiles (all >0.88). The complete average posterior probability matrix covering diagonal classification accuracy and off-diagonal cross-classification probabilities is presented in Supplementary Table S6 to transparently report classification uncertainty. All annotated R codes for the primary and sensitivity LPA analyzes are provided in the Supplementary materials.

To account for classification uncertainty inherent in latent profile assignment, we adopted posterior probability-weighted multinomial logistic regression. This analytical approach may help mitigate potential bias introduced by deterministic hard class assignment. Each participant was weighted by their maximum posterior probability (max_prob) derived from the five-class LPA solution. This analytical approach may help mitigate potential bias introduced by deterministic hard class assignment and reduce potential estimation bias introduced when treating modal latent class membership as an error-free observed variable. We acknowledge that this weighting approach, while reducing classification uncertainty, does not fully eliminate it. The Low eHealth Literacy profile (Class 1) was set as the reference group, and odds ratios (ORs) with 95% confidence intervals (CIs) were estimated to quantify covariate associations with profile membership. Given the sparse sample of retired participants, we aggregated retired, student, and other unemployed respondents into a unified non-employed category to avoid quasi-complete separation and unstable parameter estimation, with employed participants treated as the reference group. We constructed a fully adjusted model incorporating age, gender, education, household income, residential location, chronic disease status, medical insurance coverage, and employment category. Several supplementary sensitivity analyzes were further performed to examine the consistency of observed cross-sectional associations, including substituting continuous age with categorical age groups, excluding the employment covariate, and re-estimating the weighted regression after removing all retired participants. Variance inflation factor analysis was conducted to detect multicollinearity (mean VIF = 1.45), and a quadratic age term was included to examine the linearity assumption of age effects (*p* = 0.737). All regression models were consistently weighted by posterior probabilities, and full analytical codes and detailed outputs are provided in the Supplementary materials.

### 2.4 Ethical considerations

This study’s protocol was reviewed and approved by the Ethics Committee of Jining Medical University (Approval No.: JNMC-2022-YX-013). The ethics committee granted a waiver of written informed consent consistent with the online anonymous survey format; all participants provided electronic informed consent before filling out the questionnaire.

## 3 Results

### 3.1 Participant characteristics

A total of 1,391 valid participants were included in the final analysis, with a mean age of (37.9 ± 13.0) years. The detailed demographic and health-related characteristics of the participants are presented in Table 1. Briefly, 946 (68.0%) were female; 782 (56.2%) were urban residents; 913 (65.6%) had college or above educational level; 1,071 (77.0%) were employed; 318 (22.9%) had commercial insurance; 237 (17.0%) had chronic diseases; 503 (36.2%) had an annual household income of less than 50,000 RMB. See Table 1 for more details.

**Table 1:** Overall demographic characteristics *N* (%).

Characteristic	*N* = 1,391^1^
Gender	
Female	946.0 (68.0)
Male	445.0 (32.0)
Residential location	
Urban	782.0 (56.2)
Rural	609.0 (43.8)
Age (years)	37.9 (13.0)
Education level	
Primary and below	158.0 (11.4)
Junior school	183.0 (13.2)
High school	137.0 (9.8)
College degree and above	913.0 (65.6)
Employment status	
Employed	1071.0 (77.0)
Non-employed	320.0 (23.0)
Commercial medical insurance status	
Insured	318.0 (22.9)
Uninsured	1073.0 (77.1)
Chronic disease status	
With chronic disease	237.0 (17.0)
Without chronic disease	1154.0 (83.0)
Annual household income (CNY)	
Less than 50,000	503.0 (36.2)
50,000–99,999	364.0 (26.2)
100,000–149,999	246.0 (17.7)
150,000–199,999	107.0 (7.7)
200,000 and above	171.0 (12.3)

1 *n* (%); mean (SD).

### 3.2 Latent profiles of electronic health literacy

Based on predefined fit criteria and two targeted sensitivity analyzes addressing potential bias arising from the single-item decision-making dimension, the 5-class latent profile solution exhibited consistent empirical performance across analytical tests and was retained for subsequent cross-sectional association analyzes. The optimal profile number was determined comprehensively based on statistical fit performance, classification accuracy, subgroup adequacy, theoretical interpretability, and substantive practical significance. As presented in Table 2, the 5-class model produced the lowest AIC (7642.00) and BIC (7757.49), the highest entropy (0.952), and a statistically significant BLRT result (*p* < 0.001), indicating superior model fit and reliable individual classification. The 6-class model (from the second sensitivity analysis excluding the decision-making dimension) showed a lower BIC (5901) but was discarded because it contained an extremely small subgroup constituting only 1.58% of the sample, indicating high overfitting risk. While the 4-class model offers greater parsimony, it fails to separate two practically meaningful unbalanced eHealth literacy subgroups. The five-profile solution captures heterogeneous patterns of uneven digital health competency across the three theoretical dimensions, with all subgroups having a reasonable sample size (minimum *n* = 137, >9.8% of total participants), and provides refined evidence for targeted health promotion strategies. The diagonal average posterior probabilities for the five profiles were 0.538, 0.882, 0.902, 0.909, and 0.952, with overall entropy of 0.952. While four profiles exceeded the conventional 0.70 threshold, Class 1 (Low eHealth Literacy) fell below this benchmark (0.538). The complete posterior probability matrix is presented in Supplementary Table S6. Thus, the 5-class solution was finally selected as the optimal latent profile model. Accordingly, five distinct eHealth literacy latent profiles were extracted, and their descriptive statistics are summarized in Table 3.

**Table 2:** Fit indices of LPA for different classes of profiles (1–6 classes).

Classes	AIC	BIC	Entropy	Prob_min	Prob_max	N_min	N_max	BLRT_p
1	11851.00	11882.89	1.000	1.000	1.000	1,391	1,391	—
2	10193.00	10245.01	0.853	0.930	0.974	445	946	<0.001
3	9327.00	9399.91	0.864	0.928	0.953	289	545	<0.001
4	8440.00	8533.85	0.933	0.940	0.981	98	579	<0.001
**5**	**7642.00**	**7757.49**	**0.952**	**0.830**	**1.000**	**137**	**430**	**<0.001**
6	7650.33	7786.51	0.839	0.0223	1.000	8	427	>0.05

AIC, Akaike Information Criterion; BIC, Bayesian Information Criterion; Prob_min, minimum posterior probability; Prob_max, maximum posterior probability; N_min, minimum sample size of latent subgroups; N_max, maximum sample size of latent subgroups; BLRT_*p*, *p*-value of Bootstrap Likelihood Ratio Test. Smaller AIC and BIC values indicate better model fit; entropy >0.7 represents acceptable classification reliability. The optimal 5-class model is bolded.

**Table 3:** Dimension scores of five latent profile groups (M ± SD).

Latent profile ID	Profiles	*N* (%)	Application ability	Critical evaluation ability	Decision-making ability
Class 1	Low eHealth literacy	157 (11.29)	2.62 ± 0.90	2.34 ± 0.96	1.71 ± 0.46
Class 2	High balanced eHealth literacy	413 (29.69)	4.72 ± 0.60	4.80 ± 0.49	5.00 ± 0.00
Class 3	Moderate-high balanced eHealth literacy	430 (30.91)	3.95 ± 0.47	3.95 ± 0.44	4.00 ± 0.00
Class 4	Moderate-low eHealth literacy	254 (18.26)	3.17 ± 0.51	3.03 ± 0.40	3.00 ± 0.00
Class 5	Application-preferred moderate-high eHealth literacy	137 (9.85)	4.24 ± 0.38	3.99 ± 0.43	3.00 ± 0.00
Full sample	Total	1,391 (100)	3.87 ± 0.91	3.82 ± 0.97	3.65 ± 1.07

All indicators were measured on a 5-point Likert scale; higher scores represent higher eHealth literacy.

**Figure 2:**
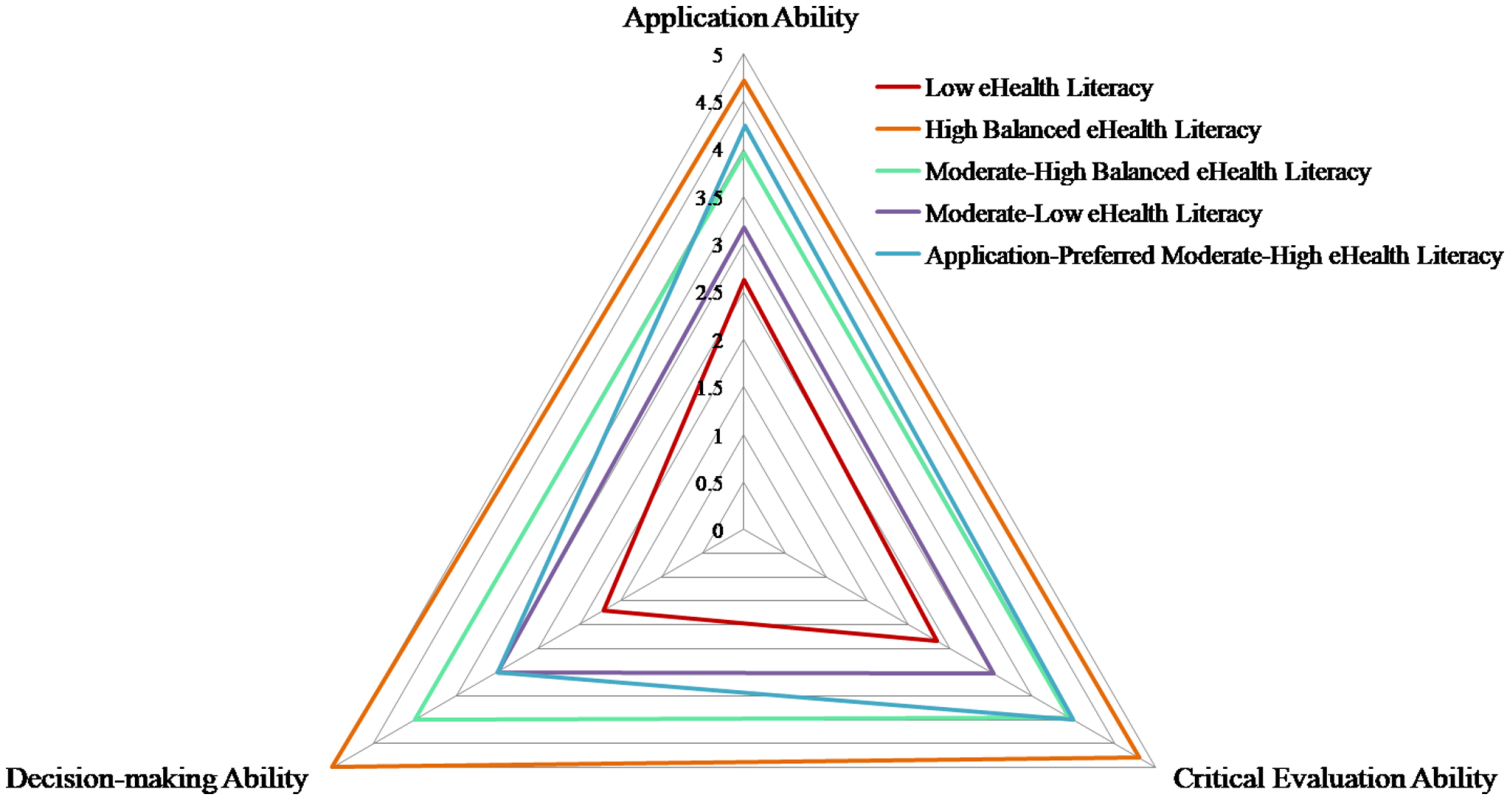
Radar chart of the three dimensions of eHealth literacy across five latent profiles.

Each profile was labeled according to its unique scoring pattern across the three dimensions: Low eHealth Literacy (*n* = 157, 11.29%): This group obtained the lowest scores across all three dimensions (Application: 2.62 ± 0.90; Critical Evaluation: 2.34 ± 0.96; Decision-Making: 1.71 ± 0.46). Moderate-Low eHealth Literacy (*n* = 254, 18.26%): All three dimensions stayed at a moderate-low level (Application: 3.17 ± 0.51; Critical Evaluation: 3.03 ± 0.40; Decision-Making: 3.00 ± 0.00). Moderate-High Balanced eHealth Literacy (*n* = 430, 30.91%): Scores of the three dimensions were evenly distributed at a moderate-high level (Application: 3.95 ± 0.47; Critical Evaluation: 3.95 ± 0.44; Decision-Making: 4.00 ± 0.00), showing balanced digital health capacity. Application-Preferred Moderate-High eHealth Literacy (*n* = 137, 9.85%): Participants performed well on information application (4.24 ± 0.38) and critical evaluation (3.99 ± 0.43) but scored only 3.00 in decision-making, forming an imbalanced profile. High Balanced eHealth Literacy (*n* = 413, 29.69%): This subgroup achieved the highest scores on all three indicators (Application: 4.72 ± 0.60; Critical Evaluation: 4.80 ± 0.49; Decision-Making: 5.00 ± 0.00), with comprehensive competence in searching, judging and utilizing online health information. In addition, Figure 2 shows the dimension scores of the five different profiles.

### 3.3 Sociodemographic correlates of latent eHealth literacy profiles

Weighted multinomial logistic regression models were fitted to identify correlates of latent eHealth literacy profiles. Each participant was weighted by their maximum posterior probability from the five-class LPA to mitigate classification uncertainty. Latent Profile Class 1 (the Low eHealth Literacy subgroup) was designated as the fixed reference category; all reported odds ratios quantify the relative odds of belonging to Class 2, Class 3, Class 4 or Class 5 compared with this Low eHealth Literacy group. The full analytical sample contained 1,391 respondents. Model diagnostics indicated no severe multicollinearity (mean VIF = 1.45). A quadratic age term was incorporated to test the linearity assumption for chronological age, and the test result (*p* = 0.737) supported a linear age association. Given the limited sample of retired individuals, retired, student and unemployed participants were combined into a unified non-employed group to avoid unstable parameter estimates caused by sparse cells. Fully adjusted odds ratios (OR) and corresponding 95% confidence intervals (CIs) are presented in Table 4.

**Table 4:** Weighted multinomial logistic regression analyzes of correlates for latent eHealth literacy profiles.

Covariates	Class 2	*p*-value	Class 3	*p*-value	Class 4	*p*-value	Class 5	*p*-value
Gender: male (reference group: Female)	1.131 (0.760, 1.684)	0.542	0.969 (0.649, 1.446)	0.877	0.747 (0.478, 1.166)	0.198	0.967 (0.585, 1.599)	0.897
Education level (reference group: Primary school and below)								
Junior high school	1.562 (0.719, 3.394)	0.260	1.233 (0.555, 2.740)	0.606	1.659 (0.725, 3.799)	0.231	**3.774 (1.506, 9.452)**	0.005**
High school	0.666 (0.319, 1.390)	0.278	1.140 (0.553, 2.349)	0.722	0.685 (0.304, 1.542)	0.346	**3.895 (1.687, 8.996)**	0.001**
College and above	0.729 (0.337, 1.575)	0.420	1.028 (0.478, 2.210)	0.944	0.539 (0.235, 1.234)	0.143	**4.552 (1.880, 11.023)**	0.001**
Annual household income (reference group: 200,000 CNY and above)								
Less than 50,000 CNY	1.674 (0.923, 3.033)	0.089	1.663 (0.917, 3.015)	0.094	1.114 (0.585, 2.121)	0.743	**3.107 (1.475, 6.542)**	0.003**
50,000–99,999 CNY	1.695 (0.735, 3.908)	0.216	2.053 (0.892, 4.727)	0.091	0.886 (0.340, 2.308)	0.804	**3.090 (1.122, 8.506)**	0.029*
100,000–149,999 CNY	1.748 (0.868, 3.521)	0.116	1.477 (0.735, 2.968)	0.274	0.793 (0.368, 1.709)	0.555	**4.995 (2.126, 11.742)**	<0.001**
150,000–199,999 CNY	0.979 (0.610, 1.571)	0.930	1.034 (0.648, 1.649)	0.890	0.739 (0.450, 1.214)	0.233	1.574 (0.836, 2.961)	0.159
Chronic disease status (reference group: No chronic disease)	**0.556 (0.325, 0.952)**	0.033*	0.902 (0.540, 1.505)	0.692	0.703 (0.391, 1.262)	0.238	**3.408 (1.920, 6.050)**	<0.001**
Commercial medical insurance coverage (reference group: Uninsured)	1.119 (0.681, 1.839)	0.657	1.282 (0.786, 2.091)	0.321	0.811 (0.462, 1.424)	0.466	**2.060 (1.187, 3.576)**	0.010*
Residential type (reference group: Rural residence)	0.655 (0.426, 1.008)	0.054	0.727 (0.477, 1.110)	0.140	0.707 (0.449, 1.115)	0.136	**0.579 (0.340, 0.986)**	0.044*
Employment status (reference group: Employed)	1.009 (0.656, 1.551)	0.967	**0.535 (0.340, 0.842)**	0.007**	1.142 (0.726, 1.798)	0.567	0.740 (0.416, 1.315)	0.306
Age (continuous variable, unit: years)	**0.977 (0.958, 0.997)**	0.026*	**0.952 (0.933, 0.972)**	<0.001**	0.985 (0.963, 1.008)	0.189	**0.936 (0.910, 0.962)**	<0.001**

Latent Profile Class 1 (low eHealth literacy subgroup) was set as the reference group for all comparisons. To account for classification uncertainty from latent profile assignment, each observation was weighted by its maximum posterior probability derived from the five-class latent profile analysis. All categorical covariates adopted the following reference groups: female gender, primary school and below education, annual household income ≥200,000 CNY, no chronic disease, no commercial medical insurance, rural residence, and employed status. Occupational categories were merged into employed and non-employed groups to address sparse-cell bias among retired participants and avoid quasi-complete separation. Variance inflation factor diagnostics confirmed no severe multicollinearity across all predictors (mean VIF = 1.45; all VIFs <2.24). The linearity assumption for continuous age was verified via a quadratic age term (*p* = 0.737). Two-tailed *p*-values are presented. *p < 0.05, **p < 0.01. Bolded values are statistically significant (*p* < 0.05, *p < 0.01).

Correlates of membership in Class 2 (High Balanced eHealth Literacy).

Relative to participants in the Low eHealth Literacy reference group (Class 1), two covariates showed statistically significant associations with belonging to the High Balanced profile (Class 2). Participants with chronic disease had significantly **lower** odds of belonging to Class 2 versus the Low eHealth Literacy reference group (OR = 0.556, 95% CI: 0.325–0.952, *p* = 0.033). Urban residents had significantly lower odds of membership to Class 2 versus the Low eHealth Literacy reference group (Class 1) (OR = 0.655, 95% CI: 0.426–1.008, *p* = 0.054). Age showed a marginally negative trend association with Class 2 membership relative to the Low eHealth Literacy reference group, that each one-year increase in age was associated with reduced odds of Class 2 membership (OR = 0.977, 95% CI: 0.958–0.997, *p* = 0.026). Gender, education, household incomeand commercial medical insurance exhibited no significant correlations with the likelihood of being classified into Class 2 rather than the Low eHealth Literacy reference group (all *p* > 0.05).

Correlates of membership in Class 3 (Moderate-High Balanced eHealth Literacy).

Two covariates displayed statistically significant cross-sectional correlations with Class 3 membership relative to the Low eHealth Literacy reference group (Class 1). Non-employed participants had significantly lower odds of belonging to Class 3 compared with employed participants in the Low eHealth Literacy reference group (Class 1) (OR = 0.535, 95% CI: 0.340–0.842, *p* = 0.007). Each one-year increase in age was associated with reduced odds of membership in Class 3 versus the Low eHealth Literacy reference group (Class 1) (OR = 0.952, 95% CI: 0.933–0.972, *p* < 0.001). No other sociodemographic, socioeconomic or health-related covariates were significantly correlated with the probability of being assigned to Class 3 relative to the Low eHealth Literacy reference group (Class 1) (all *p* > 0.05).

Correlates of membership in Class 4 (Moderate-Low eHealth Literacy).

None of the examined covariates were significantly associated with membership in the moderate-low balanced subgroup (Class 4) relative to the Low eHealth Literacy reference profile (Class 1) (all *p* > 0.05).

Correlates of membership in Class 5 (Application-Preferred Moderate-High eHealth Literacy).

Multiple socioeconomic and health-related characteristics displayed significant cross-sectional correlations with the odds of being classified into Class 5 relative to the Low eHealth Literacy reference group (Class 1).

(1) Educational attainment. Relative to participants with primary school education or below (the reference education subgroup), respondents with junior high school (OR = 3.774, 95% CI: 1.506–9.452, *p* = 0.005), high school (OR = 3.895, 95% CI: 1.687–8.996, *p* = 0.001), and college or above education (OR = 4.552, 95% CI: 1.880–11.023, *p* = 0.001) had significantly higher odds of membership in Class 5 instead of the Low eHealth Literacy reference group (Class 1).
(2) Household income. With households reporting an annual income of 200,000 CNY and above set as the reference category, participants in income brackets below 150,000 CNY showed significantly greater odds of belonging to Class 5 relative to the Low eHealth Literacy reference subgroup (Class 1): annual income below 50,000 CNY (OR = 3.107, 95% CI: 1.475–6.542, *p* = 0.003), 50,000–99,999 CNY (OR = 3.090, 95% CI: 1.122–8.506, *p* = 0.029), and 100,000–149,999 CNY (OR = 4.995, 95% CI: 2.126–11.742, *p* < 0.001). No statistically significant cross-sectional correlation with Class 5 versus Class 1 was identified for respondents in the 150,000–199,999 CNY income bracket (OR = 1.574, 95% CI: 0.836–2.961, *p* = 0.159). No significant income-related associations were detected between any income bracket and membership in Class 2, Class 3, or Class 4 when each was compared against the Class 1 reference group.
(3) Health and residential variables. Individuals living with chronic disease had significantly higher odds of Class 5 membership relative to the Low eHealth Literacy reference group (Class 1) (OR = 3.408, 95% CI: 1.920–6.050, *p* < 0.001). Participants covered by commercial medical insurance also presented greater likelihood of this profile versus the Low eHealth Literacy reference group (Class 1) (OR = 2.060, 95% CI: 1.187–3.576, *p* = 0.010). Urban residents showed significantly lower odds of being classified into Class 5 compared with rural residents in the Low eHealth Literacy reference group (Class 1) (OR = 0.579, 95% CI: 0.340–0.986, *p* = 0.044). Each additional year of age was associated with a significant reduction in the odds of Class 5 membership relative to the Low eHealth Literacy reference group (Class 1) (OR = 0.936, 95% CI: 0.910–0.962, *p* < 0.001). Gender and employment status showed no significant associations with the probability of Class 5 membership versus the Low eHealth Literacy reference group (Class 1) (all *p* > 0.05).

The diagonal average posterior probabilities for the five profiles were 0.538, 0.882, 0.902, 0.909, and 0.952. Notably, Class 1 (Low eHealth Literacy) exhibited a diagonal probability of 0.538, which is below the conventional 0.70 threshold specified in our model-selection criteria. This indicates that participants assigned to this profile have relatively lower classification certainty compared with the other four profiles, a limitation that warrants consideration when interpreting regression results that use Class 1 as the reference category. To assess the robustness of the regression results given the lower posterior classification quality of the reference profile (Class 1, diagonal posterior = 0.538), we conducted two supplementary robustness checks (Supplementary Table S9). First, we compared the primary weighted model with an unweighted multinomial regression model that treated LPA assignment as deterministic; the odds ratios for all core associations differed by less than 10% between the two specifications, and the direction and statistical significance of all predictors remained unchanged. Second, we re-estimated the weighted model using Class 4 (Moderate-Low Balanced, diagonal posterior = 0.909) as an alternative reference category. Class 4 was selected because it has the highest classification certainty among all profiles, a theoretically neutral scoring pattern, and an adequate sample size (*n* = 254, 18.26%). In this alternative specification, the direction and significance of all core associations for Class 5 remained consistent with the primary model: higher education (OR range: 2.27–8.45, all *p* < 0.05), chronic disease (OR = 4.85, *p* < 0.001), commercial insurance (OR = 2.54, *p* < 0.001), and age (OR = 0.95, *p* < 0.001) all remained significant predictors. Urban residence was not significant in this specification (OR = 0.82, *p* > 0.05), which is consistent with its marginal status in the primary model (*p* = 0.044). These supplementary analyzes confirm that the main correlational patterns are robust and not driven by the lower classification certainty of the reference profile.

### 3.4 Supplementary analysis

Two sets of supplementary analyzes were conducted to examine the consistency of cross-sectional associations derived from the primary regression models and explore potential confounding bias. First, we compared crude age-only regression and the fully adjusted weighted multinomial logistic model to examine the age association with Class 5 (Application-Preferred Moderate-High eHealth Literacy). The unadjusted single-variable model yielded a non-significant positive correlation between age and Class 5 membership (OR = 1.003, 95% CI: 0.987–1.019, *p* = 0.750). After controlling for all sociodemographic and health covariates, age showed a statistically significant negative association (OR = 0.936, 95% CI: 0.910–0.962, *p* < 0.001). This contrasting result suggested a statistical suppression effect, in which socioeconomic and health-related confounding variables masked the observed inverse cross-sectional association between age and the application-oriented high literacy profile seen in univariate analysis. Corresponding crude and fully adjusted regression outputs are available in Supplementary materials.

Second, three distinct sensitivity tests were implemented to assess result stability: substituting continuous age with categorical age groups, excluding employment status from the regression equation, and re-running the full model after removing all retired participants from the analytical sample. Across all three alternative model specifications, the direction, magnitude, and statistical significance of core correlates remained consistent with those observed in the main analytical model, which offered empirical support for the consistency of our cross-sectional findings. Detailed results of these sensitivity analyzes are presented in Supplementary Tables S7 and S8. The fully annotated Stata command syntax and complete raw analytical log outputs are provided within the supplementary text file to facilitate replication.

## 4 Discussion

### 4.1 Principal findings

This cross-sectional study adopted LPA and posterior probability-weighted multinomial logistic regression to identify distinct eHealth literacy subgroups among community residents and their cross-sectional correlates. Given the observational study design, all detected relationships are merely concurrent statistical associations; no causal inferences can be drawn from these cross-sectional data. Key principal findings are elaborated below, with tentative, hypothesis-generating interpretations aligned with available data and constraints noted throughout.

Five distinct subgroups were delineated based on three eHealth dimensions (information application, critical evaluation, health decision-making), revealing clear divergent scoring patterns that illustrate meaningful within-sample heterogeneity. Low eHealth Literacy (*n* = 157, 11.29%): Consistently lowest scores across all three dimensions (Application: 2.62 ± 0.90; Critical Evaluation: 2.34 ± 0.96; Decision-Making: 1.71 ± 0.46), showing limited capacity to source, assess and act on online health content. Moderate-Low Balanced eHealth Literacy (*n* = 254, 18.26%): Participants in this subgroup showed uniform moderate-low scores across all three measured digital health dimensions. Moderate-High Balanced eHealth Literacy (*n* = 430, 30.91%): The largest subgroup with evenly matched moderate-high scores for application and evaluation and a fixed decision-making score of 4.00, representing the most prevalent balanced literacy pattern in the sample. High Balanced eHealth Literacy (*n* = 413, 29.69%): Near-maximum scores across all dimensions (Application: 4.72 ± 0.60; Critical Evaluation: 4.80 ± 0.49; Decision-Making: 5.00 ± 0.00). However, the concentration of scores at the upper bound of the Likert scale raises potential ceiling effects and self-report response biases that warrant consideration when interpreting this profile. Finally, the Application-Preferred Moderate-High eHealth Literacy subgroup (*n* = 137, 9.85%) demonstrated strong application and evaluation scores but a fixed low decision-making score (3.00), forming an unbalanced digital competency profile that may reflect a distinct pattern of self-perceived eHealth literacy.

### 4.2 Analysis of subgroup characteristics based on regression results

Weighted multinomial logistic regression revealed that educational attainment, household income, age, residential location, employment status, chronic disease, and commercial medical insurance exhibited distinct cross-sectional correlations with different eHealth literacy latent profiles; gender showed no statistical link with profile membership. All observed statistical links are interpreted solely as concurrent correlational patterns, not causal relationships.

The regression model incorporated each participant’s maximum posterior probability to reduce LPA classification bias, with Class 1 (Low eHealth Literacy) set as the reference group. Diagnostic tests ruled out severe multicollinearity (mean VIF = 1.45) and verified a linear relationship between age and profile membership (quadratic age term *p* = 0.737). Retired, student and unemployed respondents were merged into a unified non-employed group to prevent biased estimates from sparse subgroups. However, it should be noted that Class 1 had a diagonal average posterior probability of 0.538, below the conventional 0.70 threshold. Therefore, the odds ratios reported below, which are interpreted relative to this profile, should be considered with this classification uncertainty in mind. To assess the robustness of the findings, we re-estimated the model using Class 4 (Moderate-Low Balanced; diagonal posterior = 0.909) as an alternative reference category; the direction and statistical significance of all core associations remained unchanged (Supplementary Table S9), suggesting that the main correlational patterns are stable despite the lower certainty of Class 1. Previous studies have shown that eHealth literacy decreases with increasing age and is correlated with socioeconomic characteristics (24, 25), findings that are corroborated by the present results. For Class 2 (High Balanced eHealth Literacy), two covariates showed significant associations: chronic disease (OR = 0.556, *p* = 0.033) and age (OR = 0.977, *p* = 0.026), both negatively correlated with membership in this profile. Urban residence showed a marginally negative trend (OR = 0.655, *p* = 0.054), while all other sociodemographic and health variables produced non-significant results. The negative age association aligns with prior reports (24, 25), suggesting that older adults are less likely to exhibit comprehensively high eHealth literacy.

Although some studies have indicated that women have higher eHealth literacy levels (26–29), no gender-related association was observed in the current sample. Given the absence of measures capturing gendered social roles or health decision-making behaviours, we refrain from speculative gender-related interpretations. Two significant cross-sectional correlates were identified for Class 3 (Moderate-High Balanced eHealth Literacy): non-employment (OR = 0.535, *p* = 0.007) and advancing age (OR = 0.952, *p* < 0.001). These patterns are consistent with prior studies reporting a negative association between age and eHealth literacy (24, 25), indicating that older and non-working participants exhibited lower odds of belonging to this balanced moderate-to-high literacy subgroup. Given the cross-sectional design, we do not infer causality and suggest that future longitudinal research may explore whether workplace digital health engagement or age-related digital barriers underlie these observed patterns. No other covariates reached statistical significance.

A series of sociodemographic and health factors were significantly associated with Class 5 (Application-Preferred Moderate-High eHealth Literacy), and we refrain from framing income and commercial medical insurance as protective factors because this survey failed to collect data on health management services, medical insurance benefits and income-related digital health resources, so all potential underlying correlational pathways are only tentative hypotheses waiting for subsequent verification. In line with existing research that has highlighted the important role of educational level in eHealth literacy (11, 30, 31), relative to participants with primary school education or below, respondents with junior high, high school and college education all had significantly higher odds of this profile (OR range: 3.774–4.552, all *p* < 0.01). We propose higher education may be linked to stronger online information retrieval capacity, which requires longitudinal data for validation.

Notably, participants with household incomes below 150,000 CNY exhibited elevated odds of Class 5 membership compared with those in the highest income bracket (≥200,000 CNY), with ORs ranging from 3.090 to 4.995 (*p* < 0.05). This pattern does not align with the conventional assumption that higher economic status corresponds to superior health resource accessibility. One tentative explanation is that lower-income individuals may rely more heavily on free public online health resources, potentially leading to greater engagement with digital health information — a hypothesis that warrants further investigation.

Consistent with previous evidence identifying chronic disease status as one of the important determinants of eHealth literacy (12), individuals with chronic diseases also showed positive correlations with Class 5 membership (OR = 3.408, *p* < 0.001). Commercial medical insurance was also positively associated with this profile (OR = 2.060, *p* = 0.010). These findings align with health behaviour frameworks (32) suggesting that health-related needs and insurance-facilitated access may drive engagement with digital health resources. In addition, older age (OR = 0.936, *p* < 0.001) and urban living (OR = 0.579, *p* = 0.044) were negatively correlated with membership in this application-oriented subgroup, while gender displayed no significant association and no unfounded gender-related speculation is provided.

Comparisons between univariate age models and fully adjusted multivariate models revealed a statistical suppression pattern for age’s cross-sectional correlation with Class 5 membership. The crude age-only model returned a non-significant positive correlation (OR = 1.003, *p* = 0.750), whereas full covariate adjustment revealed a strong negative cross-sectional association (OR = 0.936, *p* < 0.001). This pattern indicates unmeasured socioeconomic and health confounders mask the observed age-literacy correlation in bivariate analysis, though cross-sectional data cannot untangle temporal ordering of these variables.

Three supplementary sensitivity analyzes were conducted to examine the consistency of observed cross-sectional associational patterns, including recoding age into categorical groups, removing employment as a covariate, and excluding retired participants to address sparse-cell concerns. The direction, magnitude and significance of all core odds ratios remained consistent across alternative model specifications, demonstrating the observed cross-sectional associations are not artifacts of variable coding or small occupational subgroups.

### 4.3 Implications for intervention

Given the cross-sectional observational design, the following intervention implications should be interpreted as hypothesis-generating directions for future research rather than prescriptive practice guidelines. These suggestions are grounded in the observed subgroup characteristics and correlational patterns, but their effectiveness requires empirical testing in prospective or interventional study designs.

For participants belonging to the Low and Moderate-Low balanced subgroups, the observed cross-sectional correlational patterns suggest that universal foundational digital health skill training could be examined in future trials to assess potential concurrent shifts in online navigation capacity. These two subgroups together comprise nearly 30% of the sample and exhibit consistently Low to Moderate-Low scores across all three eHealth literacy dimensions, indicating a need for basic competency building in information searching, critical appraisal, and health decision-making. Future research could examine whether such training programs effectively raise overall literacy levels and reduce between-group disparities, particularly among older and non-employed adults who showed lower odds of belonging to the higher-literacy subgroups. The Application-Preferred Moderate-High subgroup exhibited strong information searching and evaluation skills alongside limited health decision-making ability. This cross-sectional pattern forms a testable descriptive hypothesis for subsequent research to explore whether tailored health decision-making guidance coincides with more balanced digital health competency. The High Balanced subgroup constitutes the largest high-competency group in the sample (29.69%). Longitudinal research could explore whether consistent engagement with high-quality digital health content is associated with the sustained maintenance of balanced high-literacy profiles over time.

The negative association between urban residence and membership in the higher-literacy subgroups (particularly Class 2, marginally *p* = 0.054, and Class 5, *p* = 0.044) was unexpected. While speculative, this pattern raises the hypothesis that urban residents may face greater exposure to fragmented or low-quality online health information, potentially diluting the benefits of higher digital access. Future research could investigate whether urban-specific digital health literacy interventions — such as media literacy training or curated health information platforms — effectively counterbalance this observed disparity.

A cross-cutting consideration is the zero within-profile standard deviation observed for the decision-making dimension across several profiles. While this reflects the single-item nature of this measure rather than a true lack of variation, it suggests that future intervention studies may benefit from using multi-item decision-making scales to capture more nuanced changes in this dimension. Additionally, the suppression effect identified for age — where the negative age-literacy association only emerged after adjusting for education and income — implies that interventions targeting educational and economic disparities may indirectly buffer age-related digital literacy declines.

### 4.4 Strengths, limitations, and future directions

This study addresses a public health issue focused on eHealth literacy, adopting a person-centered LPA strategy to identify heterogeneous subgroups beyond conventional total-score-based research, which provides refined evidence for understanding digital health disparities among residents. All core analytical results are clearly reported in standardized tables to ensure transparency and replicability. At the same time, posterior probability-weighted multinomial logistic regression was adopted to account for latent profile classification uncertainty. Combined with multiple sensitivity tests, this approach may help improve the consistency of the cross-sectional associations observed between individual characteristics and eHealth literacy profiles.

Several methodological and design limitations restrict the interpretative scope and external generalizability of the present study, which are elaborated systematically as follows. First, selection bias due to online convenience sampling. This survey only enrolled participants with access to the internet and digital equipment, while digitally disconnected populations were excluded from our analytical sample. As Szałata et al. (33) noted, “Selection bias emerged as a major concern, as access to and familiarity with digital technologies significantly influences who participates in online surveys. Individuals with poorer health or limited digital literacy are often underrepresented.” Accordingly, the sample overrepresents residents with basic digital accessibility, and the findings cannot be generalized to fully offline community groups. Besides, the gender distribution was skewed toward female participants, a common limitation of online questionnaire surveys reported in existing research (34, 35), which weakens the representativeness of our results for male populations, as men’s health information-seeking behaviours may not be fully captured. Although multivariate regression adjusted for confounding factors, sampling bias still constrains external validity. Second, self-report measurement and ceiling effects. All three eHealth literacy dimensions were collected via self-reported Likert scales, which are susceptible to social desirability bias. Moreover, obvious ceiling effects were observed in the High Balanced eHealth Literacy subgroup, which may compress the true variation of participants’ digital health capabilities and potentially bias the LPA classification results. Third, cross-sectional design precludes causal inference. We failed to collect longitudinal temporal data, and all detected associations between sociodemographic, economic, health-related variables and eHealth literacy latent profiles only reflect contemporaneous correlations rather than causal relationships. Fourth, uncalidated three-dimensional measurement structure. The three-dimensional eHealth literacy scale adopted in this research has not undergone rigorous localized psychometric validation targeting the study population, which brings uncertainties to the construct reliability and dimensional validity supporting our LPA subgroup partitioning. The Critical Evaluation dimension included only two items, while the Decision-Making dimension consisted of just one item — failing to meet conventional item quantity criteria for deriving a robust three-factor measurement structure. Readers should interpret the profile-based results with this measurement limitation in mind. Fifth, potential model selection uncertainty and single-item measurement artifact. Although multiple statistical fit indices and two sensitivity analyzes provided empirical support for the five-profile latent structure, latent profile analysis inherently involves potential model selection uncertainty. We conducted two rigorous sensitivity analyzes—item-level LPA using all eight original eHEALS items and dimension-based LPA excluding the decision-making dimension—and both analyzes supported the five-class solution with highly consistent subgroup distributions. Nevertheless, the primary dimension-based LPA exhibited zero within-profile standard deviation for the single-item decision-making indicator across several latent classes. This implies that the identified five-profile structure, while statistically plausible, may still partially reflect the discrete nature of this single-item measurement. The current sensitivity analyzes may reduce but cannot fully rule out this potential measurement artifact. Notably, the final five-class model demonstrated good classification reliability across primary and sensitivity analyzes, with diagonal average posterior probabilities ranging from 0.538 to 0.952 across the five profiles (Supplementary Table S6). This classification certainty was attributable to the high internal consistency of the eHEALS scale, continuous dimension-level aggregation, and highly differentiated response patterns across participants, rather than analytical errors or artificially discretized variables. Even so, the potential structural impact of the discrete single-item decision-making measure cannot be completely ruled out. No independent external cohort was available to evaluate the generalizability of the five-profile grouping. Sixth, factor analytical limitations. While the two-factor CFA model exhibited superior CFI, TLI, and SRMR fit indices compared with the unidimensional structure, its relatively high RMSEA value (0.115) indicated that this alternative factor structure still did not achieve ideal model fit in the current dataset. Accordingly, we avoid overinterpreting the discriminant properties between the two multi-item subdimensions, and we treat the hypothesized three-dimensional eHealth literacy structure as theoretically grounded rather than empirically established in the present sample. The three-dimensional grouping applied for our LPA was predefined based on existing theoretical frameworks, rather than emerging from data-driven factor structure in the present sample. Seventh, sparse cells among retired participants. The small sample size of retired participants led to sparse cells in the regression analysis; thus, we combined retired individuals with students and unemployed respondents into a unified occupational category, which prevents us from conducting granular analyzes focusing on the unique correlational characteristics of retired groups. Eighth, residual classification uncertainty remains. Although we adopted posterior probability-weighted multinomial logistic regression, which may help mitigate estimation bias related to deterministic latent profile assignment, this analytical strategy may reduce but cannot completely rule out the inherent classification uncertainty of LPA. Posterior probability weighting serves only as a single analytical adjustment that may reduce classification-related bias, rather than a comprehensive three-step causal correction framework. Future studies could adopt more advanced causal correction frameworks or repeated classification validation to further minimize bias derived from latent group assignment. Ninth, the lower posterior classification quality of the reference profile warrants caution when interpreting regression results. The diagonal average posterior probability for Class 1 (Low eHealth Literacy) was 0.538, falling below the conventional 0.70 threshold. Because all multinomial regression comparisons are interpreted relative to this profile, the lower classification certainty of Class 1 introduces some degree of uncertainty into the estimated odds ratios. Although the overall entropy of the five-profile solution was high (0.952), the three-seed robustness check confirmed the stability of the five-class structure, and supplementary analyzes using Class 4 (Moderate-Low Balanced; diagonal posterior = 0.909) as an alternative reference category yielded consistent results (Supplementary Table S9), readers should still interpret the reported odds ratios with this methodological limitation in mind. Future research may consider adopting three-step latent class regression approaches (e.g., the BCH method) to further mitigate residual classification uncertainty.

Based on the above limitations, targeted improvements can be implemented in subsequent studies from multiple dimensions. First, mixed-mode sampling combining offline paper questionnaires and online electronic surveys should be adopted to recruit digitally marginalized groups excluded in this research. This would optimize sample representativeness, reduce selection bias, and help accurately estimate the prevalence of the Low eHealth Literacy among the general adult population. Second, further localized scale validation research is required to supplement sufficient measurement items for the critical evaluation and decision-making subscales, systematically test the reliability, convergent validity and discriminant validity of the three-dimensional measurement framework, and finally develop a psychometrically stable Chinese version of the multidimensional eHealth literacy scale. Third, longitudinal tracking research can be designed on the basis of the five latent profiles identified in this study. Targeted stratified intervention programs can be implemented for different subgroups to clarify the causal pathways between sociodemographic, economic and health-related factors and heterogeneous eHealth literacy patterns, as well as the long-term dynamic evolutionary rules of digital health competencies. Fourth, mixed research designs combining quantitative LPA and qualitative in-depth interviews can be adopted to deeply explore the social, cultural and individual psychological mechanisms that shape distinct eHealth literacy developmental patterns across different subgroups.

## 5 Conclusion

This study revealed marked individual heterogeneity in eHealth literacy among Chinese participants with access to digital health services. LPA identified five interpretable eHealth literacy subgroups: Low eHealth Literacy (11.29%), Moderate-Low Balanced eHealth Literacy (18.26%), Moderate-High Balanced eHealth Literacy (30.91%), High Balanced eHealth Literacy (29.69%), and Application-Preferred Moderate-High eHealth Literacy (9.85%). Using posterior probability-weighted multinomial logistic regression, we explored cross-sectional correlates of subgroup membership. Age, educational attainment, employment status, household income, chronic disease status, commercial medical insurance, and urban residence were differentially associated with distinct eHealth literacy profiles. Higher educational attainment correlated with greater odds of belonging to the Application-Preferred Moderate-High profile (OR range: 3.774–4.552), while advancing age was negatively associated with membership in the High Balanced (Class 2, OR = 0.977), Moderate-High Balanced (Class 3, OR = 0.952), and Application-Preferred Moderate-High (Class 5, OR = 0.936) profiles. Non-employment was negatively associated with the Moderate-High Balanced profile (Class3, OR = 0.535). Participants with chronic diseases (OR = 3.408) or commercial insurance (OR = 2.060) were more likely to fall into the Application-Preferred Moderate-High profile. Household income demonstrated a profile-specific correlational pattern: lower household income was only associated with increased odds of belonging to the Application-Preferred Moderate-High subgroup versus the Low eHealth Literacy reference group (OR range: 3.090–4.995).

The cross-sectional observational results supply descriptive empirical data to characterize vulnerable groups with limited digital health capacity. All intervention-related suggestions should be interpreted as hypothesis-generating directions for future prospective research. The observed correlational distributions can serve as preliminary descriptive reference when designing subsequent digital health promotion frameworks aimed at uneven digital health access across population groups. However, given that the Low eHealth Literacy reference group (Class 1) exhibited a diagonal posterior probability below the conventional 0.70 threshold, the reported odds ratios should be interpreted with this classification uncertainty in mind.

## Data Availability

The raw data supporting the conclusions of this article will be made available by the authors, without undue reservation.

## Footnotes

1
https://www.wjx.cn/
