# Supplementary material for: Latent profile analysis of eHealth literacy and its sociodemographic correlates: a cross-sectional study
Source: Front Public Health. 2026 Jul 27;14:1904244. doi: 10.3389/fpubh.2026.1904244 (PMC13454114; doi:10.3389/fpubh.2026.1904244)
Supplement: Supplementary file 1 [file Data_Sheet_1.PDF]

```
#
================================================================================
# FINAL CODE – Robust posterior extraction using mclust with class mean matching
#
================================================================================
library(tidyLPA)
library(dplyr)
library(readxl)
library(mclust)

file_path <- "E:/ssci 教学研究论文/电子健康素养/论文 写/论文返修 0622/0623lap.xls"

# Helper: match mclust class order to tidyLPA classes via mean similarity
match_classes <- function(scaled_data, class_ids, n_classes = 5) {
  # Compute class means from the scaled data using tidyLPA classification
  tidy_means <- scaled_data %>%
    mutate(class = class_ids) %>%
    group_by(class) %>%
    summarise(across(everything(), mean)) %>%
    ungroup() %>%
    arrange(class) %>%
    select(-class) %>%              # matrix of class means (n_classes x p)
    as.matrix()

  # Fit mclust with same model (EII = model 1 in tidyLPA)
  set.seed(123)
  mclust_fit <- Mclust(scaled_data, G = n_classes, modelNames = "EII")
  z <- mclust_fit$z                 # n x n_classes, columns in mclust order
  mclust_means <- mclust_fit$parameters$mean    # p x n_classes

  # Compute distance between each tidy mean and each mclust mean
  dist_mat <- matrix(NA, n_classes, n_classes)
  for (i in 1:n_classes) {
    for (j in 1:n_classes) {
      dist_mat[i, j] <- sum((tidy_means[i, ] - mclust_means[, j])^2)
    }
  }
  # For each tidy class, find the closest mclust class
  mapping <- apply(dist_mat, 1, which.min)
  # Reorder z columns so that column i corresponds to tidy class i
  z_reordered <- z[, mapping, drop = FALSE]
  colnames(z_reordered) <- paste0("prob_", 1:n_classes)

  return(z_reordered)
```

```
}

#
==============================================================================
# Section 1: Primary Analysis (3 dimensions)
#
==============================================================================
cat("\n==================== PRIMARY ANALYSIS ====================\n")
df <- read_excel(file_path)
lpa_data <- df[, c("yingyong", "pingpan", "juece")]
lpa_complete <- na.omit(lpa_data)
lpa_scaled <- scale(lpa_complete) %>% as.data.frame()
colnames(lpa_scaled) <- c("yingyong", "pingpan", "juece")

set.seed(123)
prof_1_6 <- estimate_profiles(lpa_scaled, 1:6, 1, 500, 1e-6)
print(get_fit(prof_1_6))
compare_solutions(prof_1_6)

# Get 5-class assignments
res_all <- get_data(prof_1_6)
res_opt <- res_all %>% filter(classes_number == 5) %>% slice(1:nrow(lpa_complete))
lpa_complete$class_id <- res_opt$Class

# Extract matched posterior
z_primary <- match_classes(lpa_scaled, lpa_complete$class_id, 5)
lpa_complete <- cbind(lpa_complete, z_primary)
lpa_complete$max_prob <- apply(z_primary, 1, max)

# Average posterior matrix
post_means_primary <- lpa_complete %>%
  group_by(class_id) %>%
  summarise(across(starts_with("prob_"), mean, .names = "avg_{.col}"), n = n()) %>%
  ungroup()
cat("\n===== Average Posterior Probability Matrix (Primary) =====\n")
print(post_means_primary)

diag_primary <- post_means_primary %>%
  rowwise() %>%
  mutate(diag = get(paste0("avg_prob_", class_id))) %>%
  select(class_id, diag)
cat("\nDiagonal averages:\n")
print(diag_primary)
```

```
write.csv(lpa_complete, "LPA_5class_Primary_Analysis.csv", row.names = FALSE)

#
================================================================================
# Section 2: Sensitivity 1 (8 items)
#
================================================================================
cat("\n\n===================== SENSITIVITY 1 (8 ITEMS) =====================\n")
ITEM_COLS <- c("item1", "item2", "item3", "item4", "item5", "item6", "item7", "item8")
item_data <- df[, ITEM_COLS]
item_complete <- na.omit(item_data)
item_scaled <- scale(item_complete) %>% as.data.frame()

set.seed(123)
prof_item <- estimate_profiles(item_scaled, 1:6, 1, 500, 1e-6)
print(get_fit(prof_item) %>% select(Classes, AIC, BIC, Entropy, BLRT_p, n_min))
compare_solutions(prof_item)

res_item <- get_data(prof_item)
res_item_opt <- res_item %>% filter(classes_number == 5) %>% slice(1:nrow(item_complete))
item_complete$class_id <- res_item_opt$Class

# Extract matched posterior (using same helper)
z_item <- match_classes(item_scaled, item_complete$class_id, 5)
item_complete <- cbind(item_complete, z_item)
item_complete$max_prob <- apply(z_item, 1, max)

post_means_item <- item_complete %>%
  group_by(class_id) %>%
  summarise(across(starts_with("prob_"), mean, .names = "avg_{.col}"), n = n()) %>%
  ungroup()
cat("\n===== Average Posterior Probability Matrix (Sensitivity 1) =====\n")
print(post_means_item)

write.csv(item_complete, "LPA_5class_Sensitivity1_ItemLevel.csv", row.names = FALSE)

#
================================================================================
# Section 3: Sensitivity 2 (exclude decision dimension)
#
================================================================================
cat("\n\n===================== SENSITIVITY 2 (2 DIMENSIONS) =====================\n")
sens2_data <- df[, c("yingyong", "pingpan")]
sens2_complete <- na.omit(sens2_data)
```

```
sens2_scaled <- scale(sens2_complete) %>% as.data.frame()
colnames(sens2_scaled) <- c("yingyong", "pingpan")

set.seed(123)
prof_sens2 <- estimate_profiles(sens2_scaled, 1:6, 1, 500, 1e-6)
print(get_fit(prof_sens2) %>% select(Classes, AIC, BIC, Entropy, BLRT_p, n_min))
compare_solutions(prof_sens2)

res_sens2 <- get_data(prof_sens2)
res_sens2_opt <- res_sens2 %>% filter(classes_number == 5) %>% slice(1:nrow(sens2_complete))
sens2_complete$class_id <- res_sens2_opt$Class

# Extract matched posterior
z_sens2 <- match_classes(sens2_scaled, sens2_complete$class_id, 5)
sens2_complete <- cbind(sens2_complete, z_sens2)
sens2_complete$max_prob <- apply(z_sens2, 1, max)

post_means_sens2 <- sens2_complete %>%
  group_by(class_id) %>%
  summarise(across(starts_with("prob_"), mean, .names = "avg_{.col}"), n = n()) %>%
  ungroup()
cat("\n===== Average Posterior Probability Matrix (Sensitivity 2) =====\n")
print(post_means_sens2)

write.csv(sens2_complete, "LPA_5class_Sensitivity2_ExcludeDecision.csv", row.names = FALSE)

#
===========================================================================
# SUMMARY
#
===========================================================================
cat("\n\n==================== SUMMARY ====================\n")
cat("\nPrimary diagonal averages:\n")
print(diag_primary)

cat("\nSensitivity 1 diagonal:\n")
print(post_means_item %>% rowwise() %>% mutate(diag = get(paste0("avg_prob_", class_id))) %>% select(class_id, diag))

cat("\nSensitivity 2 diagonal:\n")
print(post_means_sens2 %>% rowwise() %>% mutate(diag = get(paste0("avg_prob_", class_id))) %>% select(class_id, diag))

cat("\nPer-participant maximum posterior probability (min and max):\n")
```

```
cat(sprintf("Primary: min=%.4f, max=%.4f\n", min(lpa_complete$max_prob), max(lpa_complete$max_prob)))
cat(sprintf("Sensitivity1: min=%.4f, max=%.4f\n", min(item_complete$max_prob), max(item_complete$max_prob)))
cat(sprintf("Sensitivity2: min=%.4f, max=%.4f\n", min(sens2_complete$max_prob), max(sens2_complete$max_prob)))
```
