# Supplementary material for: Latent profile analysis of eHealth literacy and its sociodemographic correlates: a cross-sectional study
Source: Front Public Health. 2026 Jul 27;14:1904244. doi: 10.3389/fpubh.2026.1904244 (PMC13454114; doi:10.3389/fpubh.2026.1904244)
Supplement: Supplementary file 2 [file Data_Sheet_2.PDF]

```
==================================================================================
> # FINAL CODE – Robust posterior extraction using mclust with class mean matching
>                                                                                  #
==================================================================================
> library(tidyLPA)
载入需要的程序包：tidySEM
Registered S3 method overwritten by 'tidyLPA':
  method     from
  print.LRT tidySEM
You can use the function citation('tidyLPA') to create a citation for the use of {tidyLPA}.
Mplus is not installed. Use only package = 'mclust' when calling estimate_profiles().

载入程序包：‘tidyLPA’

The following objects are masked from ‘package:tidySEM’:

    get_data, get_estimates, get_fit, poms

警告信息:
1: 程序包‘tidyLPA’是用 R 版本 4.4.3 来建造的
2: 程序包‘tidySEM’是用 R 版本 4.4.3 来建造的
> library(dplyr)

载入程序包：‘dplyr’

The following objects are masked from ‘package:stats’:

    filter, lag

The following objects are masked from ‘package:base’:

    intersect, setdiff, setequal, union

警告信息:
程序包‘dplyr’是用 R 版本 4.4.3 来建造的
> library(readxl)
警告信息:
程序包‘readxl’是用 R 版本 4.4.3 来建造的
> library(mclust)
                   __           __
   ____ ___  _____/ /_  _______/ /_
  / __ `__ \/ ___/ / / / / ___/ __/
 / / / / / / /__/ / /_/ (__  ) /_
/_/ /_/ /_/\___/_/\__,_/____/\__/   version 6.1.2
```

```
Type 'citation("mclust")' for citing this R package in publications.

载入程序包：‘mclust’

The following object is masked from ‘package:dplyr’:

    count

警告信息:
程序包‘mclust’是用 R 版本 4.4.3 来建造的
> file_path <- "E:/ssci 教学研究论文/电子健康素养/论文 写/论文返修 0622/0623lap.xls"
> # Helper: match mclust class order to tidyLPA classes via mean similarity
> match_classes <- function(scaled_data, class_ids, n_classes = 5) {
+   # Compute class means from the scaled data using tidyLPA classification
+   tidy_means <- scaled_data %>%
+     mutate(class = class_ids) %>%
+     group_by(class) %>%
+     summarise(across(everything(), mean)) %>%
+     ungroup() %>%
+     arrange(class) %>%
+     select(-class) %>%          # matrix of class means (n_classes x p)
+     as.matrix()
+
+   # Fit mclust with same model (EII = model 1 in tidyLPA)
+   set.seed(123)
+   mclust_fit <- Mclust(scaled_data, G = n_classes, modelNames = "EII")
+   z <- mclust_fit$z               # n x n_classes, columns in mclust order
+   mclust_means <- mclust_fit$parameters$mean    # p x n_classes
+
+   # Compute distance between each tidy mean and each mclust mean
+   dist_mat <- matrix(NA, n_classes, n_classes)
+   for (i in 1:n_classes) {
+     for (j in 1:n_classes) {
+       dist_mat[i, j] <- sum((tidy_means[i, ] - mclust_means[, j])^2)
+     }
+   }
+   # For each tidy class, find the closest mclust class
+   mapping <- apply(dist_mat, 1, which.min)
+   # Reorder z columns so that column i corresponds to tidy class i
+   z_reordered <- z[, mapping, drop = FALSE]
+   colnames(z_reordered) <- paste0("prob_", 1:n_classes)
+
+   return(z_reordered)
+ }
```

```
> #
==============================================================================
> # Section 1: Primary Analysis (3 dimensions)
> #
==============================================================================
> cat("\n==================== PRIMARY ANALYSIS ====================\n")

==================== PRIMARY ANALYSIS ====================
> df <- read_excel(file_path)
> lpa_data <- df[, c("yingyong", "pingpan", "juece")]
> lpa_complete <- na.omit(lpa_data)
> lpa_scaled <- scale(lpa_complete) %>% as.data.frame()
> colnames(lpa_scaled) <- c("yingyong", "pingpan", "juece")
> set.seed(123)
> prof_1_6 <- estimate_profiles(lpa_scaled, 1:6, 1, 500, 1e-6)
The 'variances'/'covariances' arguments were ignored in favor of the 'models' argument.
警告信息:
1: In estimate_profiles.default(lpa_scaled, 1:6, 1, 500, 1e-06) :
  Latent profile analysis assumes that all variables have a continuous measurement level. Some variables have fewer than 10 unique values, which may violate this assumption. Carefully examine the results, and avoid complex models, e.g.with free (co)variances or many classes. The variables violating this assumption are: pingpan, juece
2: In (function (data, modelName = NULL, nboot = 999, level = 0.05,    :
  some model(s) could not be fitted!
3:
One or more analyses resulted in warnings! Examine these analyses carefully: model_1_class_6
> print(get_fit(prof_1_6))
# A tibble: 6 × 20
  Model Classes LogLik parameters     n    AIC    AWE    BIC   CAIC    CLC    KIC
SABIC      ICL Entropy
  <dbl>   <int>  <dbl>      <dbl> <int>  <dbl>  <dbl>  <dbl>  <dbl>  <dbl>  <dbl>
<dbl>   <dbl>   <dbl>
1     1       1  -5920.          6  1391 11851. 11942. 11883. 11889. 11841. 11860.
11864. -11883.   1
2     1       2  -5086.         10  1391 10193. 10346. 10245. 10255. 10174. 10206.
10213. -10376.   0.853
3     1       3  -4649.         14  1391  9327.  9542.  9400.  9414.  9300.  9344.
9355.   -9605.   0.864
4     1       4  -4202.         18  1391  8440.  8716.  8534.  8552.  8405.  8461.
8477.   -8659.   0.933
5     1       5  -3799.         22  1391  7642.  7981.  7757.  7779.  7600.  7667.
7688.   -7862.   0.952
6     1       6  -3799.         26  1391  7650.  8051.  7787.  7813.  7600.  7679.
7704.   -8385.   0.839
```

```
# ℹ 6 more variables: prob_min <dbl>, prob_max <dbl>, n_min <dbl>, n_max <dbl>, BLRT_val <dbl>, BLRT_p <dbl>
> compare_solutions(prof_1_6)
Compare tidyLPA solutions:

 Model Classes BIC       Warnings
 1     1       11882.887
 1     2       10245.006
 1     3        9399.908
 1     4        8533.854
 1     5        7757.486
 1     6        7786.509 Warning

Best model according to BIC is Model 1 with 5 classes.

An analytic hierarchy process, based on the fit indices AIC, AWE, BIC, CLC, and KIC (Akogul & Erisoglu, 2017), suggests the best solution is Model 1 with 5 classes.
警告信息:
1:
One or more analyses resulted in warnings! Examine these analyses carefully: model_1_class_6
2: The solution with the maximum number of classes under consideration was considered to be the best solution according to one or more fit indices. Examine your results with care and consider estimating more classes.
> # Get 5-class assignments
> res_all <- get_data(prof_1_6)
> res_opt <- res_all %>% filter(classes_number == 5) %>% slice(1:nrow(lpa_complete))
> lpa_complete$class_id <- res_opt$Class
> # Extract matched posterior
> z_primary <- match_classes(lpa_scaled, lpa_complete$class_id, 5)
fitting ...

|===========================================================================
======================| 100%
> lpa_complete <- cbind(lpa_complete, z_primary)
> lpa_complete$max_prob <- apply(z_primary, 1, max)
> # Average posterior matrix
> post_means_primary <- lpa_complete %>%
+     group_by(class_id) %>%
+     summarise(across(starts_with("prob_"), mean, .names = "avg_{.col}"), n = n()) %>%
+     ungroup()
> cat("\n===== Average Posterior Probability Matrix (Primary) =====\n")

===== Average Posterior Probability Matrix (Primary) =====
> print(post_means_primary)
```

```
# A tibble: 5 × 7
  class_id avg_prob_1     avg_prob_2 avg_prob_3 avg_prob_4 avg_prob_5     n
     <dbl>      <dbl>          <dbl>      <dbl>      <dbl>      <dbl> <int>
1        1   5.38e- 1 0.00000000353     0.0267    0.436        0.0267   157
2        2   8.72e-11 0.882             0.0775    0.00111      0.0775   413
3        3   7.93e- 5 0.0378            0.902     0.0346       0.902    430
4        4   1.06e- 2 0.00000000415     0.0795    0.909        0.0795   254
5        5   9.64e-13 0.00241           0.952     0.0452       0.952    137
> diag_primary <- post_means_primary %>%
+     rowwise() %>%
+     mutate(diag = get(paste0("avg_prob_", class_id))) %>%
+     select(class_id, diag)
> cat("\nDiagonal averages:\n")

Diagonal averages:
> print(diag_primary)
# A tibble: 5 × 2
# Rowwise: 
  class_id  diag
     <dbl> <dbl>
1        1 0.538
2        2 0.882
3        3 0.902
4        4 0.909
5        5 0.952
> write.csv(lpa_complete, "LPA_5class_Primary_Analysis.csv", row.names = FALSE)
> # ==============================================================================
> # Section 2: Sensitivity 1 (8 items)
> # ==============================================================================
> cat("\n\n==================== SENSITIVITY 1 (8 ITEMS) ====================\n")


==================== SENSITIVITY 1 (8 ITEMS) ====================
> ITEM_COLS <- c("item1", "item2", "item3", "item4", "item5", "item6", "item7", "item8")
> item_data <- df[, ITEM_COLS]
> item_complete <- na.omit(item_data)
> item_scaled <- scale(item_complete) %>% as.data.frame()
> set.seed(123)
> prof_item <- estimate_profiles(item_scaled, 1:6, 1, 500, 1e-6)
The 'variances'/'covariances' arguments were ignored in favor of the 'models' argument.
警告信息:
In estimate_profiles.default(item_scaled, 1:6, 1, 500, 1e-06) :
```

Latent profile analysis assumes that all variables have a continuous measurement level. Some variables have fewer than 10 unique values, which may violate this assumption. Carefully examine the results, and avoid complex models, e.g.with free (co)variances or many classes. The variables violating this assumption are: item1, item2, item3, item4, item5, item6, item7, item8

```
> print(get_fit(prof_item) %>% select(Classes, AIC, BIC, Entropy, BLRT_p, n_min))
# A tibble: 6 × 6
  Classes    AIC    BIC Entropy   BLRT_p  n_min
    <int>  <dbl>  <dbl>   <dbl>    <dbl>  <dbl>
1       1 31604. 31688.   1       NA      1
2       2 25702. 25833.   0.948    0.00990 0.318
3       3 23085. 23263.   0.946    0.00990 0.241
4       4 21410. 21635.   0.952    0.00990 0.0633
5       5 20648. 20921.   0.988    0.00990 0.0834
6       6 20817. 21136.   0.912    1       0.0604
> compare_solutions(prof_item)
Compare tidyLPA solutions:

 Model Classes BIC
 1     1       31687.698
 1     2       25832.840
 1     3       23263.050
 1     4       21635.139
 1     5       20920.817
 1     6       21136.147
```

Best model according to BIC is Model 1 with 5 classes.

An analytic hierarchy process, based on the fit indices AIC, AWE, BIC, CLC, and KIC (Akogul & Erisoglu, 2017), suggests the best solution is Model 1 with 5 classes.

```
> res_item <- get_data(prof_item)
> res_item_opt <- res_item %>% filter(classes_number == 5) %>% slice(1:nrow(item_complete))
> item_complete$class_id <- res_item_opt$Class
> # Extract matched posterior (using same helper)
> z_item <- match_classes(item_scaled, item_complete$class_id, 5)
fitting ...

|===========================================================================
======================| 100%
> item_complete <- cbind(item_complete, z_item)
> item_complete$max_prob <- apply(z_item, 1, max)
> post_means_item <- item_complete %>%
+     group_by(class_id) %>%
+     summarise(across(starts_with("prob_"), mean, .names = "avg_{.col}"), n = n()) %>%
+     ungroup()
```

```
> cat("\n===== Average Posterior Probability Matrix (Sensitivity 1) =====\n")

===== Average Posterior Probability Matrix (Sensitivity 1) =====
> print(post_means_item)
# A tibble: 5 × 7
  class_id avg_prob_1 avg_prob_2 avg_prob_3 avg_prob_4 avg_prob_5     n
     <dbl>      <dbl>      <dbl>      <dbl>      <dbl>      <dbl> <int>
1        1    0.843     4.81e- 2   2.08e- 3   0.843     9.23e- 2   534
2        2    0.0194    9.81e- 1   5.49e-43   0.0194    1.11e-13   347
3        3    0.00663   2.59e-13   6.13e- 1   0.00663   2.65e- 1   127
4        4    0.973     2.65e- 2   1.62e-19   0.973     7.38e- 4   116
5        5    0.0881    1.19e- 3   3.95e- 2   0.0881    7.73e- 1   267
> write.csv(item_complete, "LPA_5class_Sensitivity1_ItemLevel.csv", row.names = FALSE)
>                                                                               #
==============================================================================
> # Section 3: Sensitivity 2 (exclude decision dimension)
>                                                                               #
==============================================================================
>     cat("\n\n====================     SENSITIVITY     2     (2     DIMENSIONS)
====================\n")


==================== SENSITIVITY 2 (2 DIMENSIONS) ====================
> sens2_data <- df[, c("yingyong", "pingpan")]
> sens2_complete <- na.omit(sens2_data)
> sens2_scaled <- scale(sens2_complete) %>% as.data.frame()
> colnames(sens2_scaled) <- c("yingyong", "pingpan")
> set.seed(123)
> prof_sens2 <- estimate_profiles(sens2_scaled, 1:6, 1, 500, 1e-6)
The 'variances'/'covariances' arguments were ignored in favor of the 'models' argument.
警告信息:
In estimate_profiles.default(sens2_scaled, 1:6, 1, 500, 1e-06) :
  Latent profile analysis assumes that all variables have a continuous measurement level. Some variables have fewer than 10 unique values, which may violate this assumption. Carefully examine the results, and avoid complex models, e.g.with free (co)variances or many classes. The variables violating this assumption are: pingpan
> print(get_fit(prof_sens2) %>% select(Classes, AIC, BIC, Entropy, BLRT_p, n_min))
# A tibble: 6 × 6
  Classes   AIC   BIC Entropy   BLRT_p  n_min
    <int> <dbl> <dbl>   <dbl>    <dbl>  <dbl>
1       1 7901. 7922.   1       NA      1
2       2 7024. 7061.   0.819    0.00990 0.279
3       3 6631. 6683.   0.820    0.00990 0.216
4       4 5993. 6061.   0.929    0.00990 0.0719
```

```
5            5 6111. 6195.      0.734    1             0.0158
6            6 5801. 5901.      0.922    0.00990 0.0158
> compare_solutions(prof_sens2)
Compare tidyLPA solutions:

 Model Classes BIC
 1         1            7921.924
 1         2            7061.151
 1         3            6683.410
 1         4            6060.643
 1         5            6194.984
 1         6            5900.775

Best model according to BIC is Model 1 with 6 classes.

An analytic hierarchy process, based on the fit indices AIC, AWE, BIC, CLC, and KIC (Akogul & Erisoglu, 2017), suggests the best solution is Model 1 with 6 classes.
警告信息:
The solution with the maximum number of classes under consideration was considered to be the best solution according to one or more fit indices. Examine your results with care and consider estimating more classes.
> res_sens2 <- get_data(prof_sens2)
> res_sens2_opt <- res_sens2 %>% filter(classes_number == 5) %>% slice(1:nrow(sens2_complete))
> sens2_complete$class_id <- res_sens2_opt$Class
> # Extract matched posterior
> z_sens2 <- match_classes(sens2_scaled, sens2_complete$class_id, 5)
fitting ...

|===========================================================================
======================| 100%
> sens2_complete <- cbind(sens2_complete, z_sens2)
> sens2_complete$max_prob <- apply(z_sens2, 1, max)
> post_means_sens2 <- sens2_complete %>%
+     group_by(class_id) %>%
+     summarise(across(starts_with("prob_"), mean, .names = "avg_{.col}"), n = n()) %>%
+     ungroup()
> cat("\n===== Average Posterior Probability Matrix (Sensitivity 2) =====\n")

===== Average Posterior Probability Matrix (Sensitivity 2) =====
> print(post_means_sens2)
# A tibble: 5 × 7
   class_id avg_prob_1 avg_prob_2 avg_prob_3    avg_prob_4       avg_prob_5         n
      <dbl>          <dbl>          <dbl>          <dbl>             <dbl>                 <dbl> <int>
```

```
1          1    9.86e- 1    2.34e-21    7.18e-10 0.0138        0.00000000261     74
2          2    7.92e-21    9.36e- 1    2.54e- 2 0.000000207 0.0384               403
3          3    7.45e-15    2.38e- 1    6.22e- 1 0.000786     0.140                22
4          4    3.90e- 2    1.88e- 6    5.58e- 2 0.843        0.0621              326
5          5    4.67e- 7    1.63e- 2    3.22e- 1 0.0413       0.620               566
> write.csv(sens2_complete, "LPA_5class_Sensitivity2_ExcludeDecision.csv", row.names = FALSE)
> #
==============================================================================
> # SUMMARY
> #
==============================================================================
> cat("\n\n==================== SUMMARY ====================\n")


==================== SUMMARY ====================
> cat("\nPrimary diagonal averages:\n")

Primary diagonal averages:
> print(diag_primary)
# A tibble: 5 × 2
# Rowwise:
  class_id  diag
     <dbl> <dbl>
1        1 0.538
2        2 0.882
3        3 0.902
4        4 0.909
5        5 0.952
> cat("\nSensitivity 1 diagonal:\n")

Sensitivity 1 diagonal:
> print(post_means_item %>% rowwise() %>% mutate(diag = get(paste0("avg_prob_", class_id))) %>% select(class_id, diag))
# A tibble: 5 × 2
# Rowwise:
  class_id  diag
     <dbl> <dbl>
1        1 0.843
2        2 0.981
3        3 0.613
4        4 0.973
5        5 0.773
> cat("\nSensitivity 2 diagonal:\n")
```

Sensitivity 2 diagonal:

```
> print(post_means_sens2 %>% rowwise() %>% mutate(diag = get(paste0("avg_prob_", class_id))) %>% select(class_id, diag))
# A tibble: 5 × 2
# Rowwise:
  class_id  diag
     <dbl> <dbl>
1        1 0.986
2        2 0.936
3        3 0.622
4        4 0.843
5        5 0.620
> cat("\nPer-participant maximum posterior probability (min and max):\n")

Per-participant maximum posterior probability (min and max):
> cat(sprintf("Primary: min=%.4f, max=%.4f\n", min(lpa_complete$max_prob), max(lpa_complete$max_prob)))
Primary: min=0.0000, max=1.0000
> cat(sprintf("Sensitivity1: min=%.4f, max=%.4f\n", min(item_complete$max_prob), max(item_complete$max_prob)))
Sensitivity1: min=0.0000, max=1.0000
> cat(sprintf("Sensitivity2: min=%.4f, max=%.4f\n", min(sens2_complete$max_prob), max(sens2_complete$max_prob)))
Sensitivity2: min=0.4306, max=1.0000
```
