# Supplementary material for: Latent profile analysis of eHealth literacy and its sociodemographic correlates: a cross-sectional study
Source: Front Public Health. 2026 Jul 27;14:1904244. doi: 10.3389/fpubh.2026.1904244 (PMC13454114; doi:10.3389/fpubh.2026.1904244)
Supplement: Supplementary file 3 [file Data_Sheet_3.PDF]

```
* ==============================================================================
* Title: Latent Profile Analysis of eHealth Literacy among Chinese Adults
* Purpose: Replicate all core regression, sensitivity and robustness analyses
* Data:    LPA_Results_With_Posteriors.xlsx (generated by R script)
* Output:  RTF tables for manuscript (Table 4, sensitivity, diagnostics, robustness)
* ==============================================================================

clear all
set more off

* ----------------------
* 0. Preliminary Settings
* ----------------------
cd "E:\ssci 教学研究论文\电子健康素养\论文 写\论文返修 0713"
ssc install estout, replace


* ----------------------
* 1. Data Import & Variable Check
* ----------------------
import excel "LPA_Results_With_Posteriors.xlsx", firstrow clear

* ----- Check existence of key variables -----
capture confirm variable yingyong pingpan juece
if _rc != 0 {
    display as error "Key variables (yingyong, pingpan, juece) missing, please check Excel column names"
    exit
}
capture confirm variable class_id
if _rc != 0 {
    display as error "Latent profile identifier class_id missing, verify column exists in R output"
    exit
}
capture confirm variable max_prob
if _rc != 0 {
    display as error "Maximum posterior probability max_prob missing, verify column exists in R output"
    exit
}

* ----- Standardize variable names consistent with manuscript -----
rename chengxiang residence
rename occupation occupation
rename age age
rename gender gender
rename education education
rename income income
rename chronic chronic
rename businsur businsur
rename yingyong application
rename pingpan evaluation
rename juece decision

* Rename latent profile and posterior probability variables to Stata standard naming
rename class_id LPA_Class_ID
rename max_prob Max_Posterior

* ----- Set reference group of outcome to 1 (Low eHealth Literacy) -----
* Recode if class_id does not start at 1 (R output class_id ranges 1~5, no recoding needed)
tab LPA_Class_ID

* ----------------------
* 2. Covariate Recoding & Generation
* ----------------------
* Occupational grouping (employed vs non-employed)
gen emp_cat = .
replace emp_cat = 1 if occupation == "employed"
replace emp_cat = 2 if inlist(occupation, "retired", "student", "others")
label define emp_lab 1 "Employed (reference)" 2 "Non-employed"
label values emp_cat emp_lab

* Age grouping for sensitivity analysis models
gen age_group = 4
```

```
replace age_group = 1 if age <= 34
replace age_group = 2 if age > 34 & age <= 49
replace age_group = 3 if age > 49 & age <= 64
label define agelab 1 "18-34" 2 "35-49" 3 "50-64" 4 "≥65"
label values age_group agelab

* Quadratic age term for linearity assumption test
gen age_sq = age^2

* Generate dummy variables (reference categories aligned with manuscript)
* Gender (female = reference 0)
tab gender, gen(gender_)
drop gender_1
rename gender_2 male

* Education attainment (primary school and below = reference 0)
tab education, gen(edu_)
rename edu_2 edu_junior
rename edu_3 edu_high
rename edu_4 edu_col
drop edu_1

* Household income (≥200,000 CNY = reference 0)
tab income, gen(inc_)
rename inc_1 inc_50less
rename inc_2 inc_50_100
rename inc_3 inc_100_150
rename inc_4 inc_150_200
rename inc_5 inc_200up


* Chronic disease status (no chronic disease = reference 0)
tab chronic, gen(chro_)
drop chro_1
rename chro_2 chronic_yes

* Private medical insurance (no private insurance = reference 0)
tab businsur, gen(ins_)
drop ins_1
rename ins_2 businsur_yes

* Residential location (rural = reference 0)
tab residence, gen(res_)
drop res_1
rename res_2 urban

* ----- Verify all dummy variables are correctly generated -----
ds male edu_* inc_* chronic_yes businsur_yes urban
tabstat `r(varlist)', by(LPA_Class_ID) statistics(mean)  // Quick descriptive overview

* ----------------------
* 3. Descriptive Statistics (Subscale Means & Sample Distribution)
* ----------------------
estpost tabstat application evaluation decision, by(LPA_Class_ID) ///
    statistics(n mean sd) columns(statistics) listwise

esttab . using "LPA_Subgroup_Stats.csv", ///
    cells("n mean sd") label replace nomtitle nonumber ///
    title("Table: Subgroup Means and Standard Deviations")

* 同时导出类别分布（频数表）
tab LPA_Class_ID, matcell(subgroup_dist)
svmat subgroup_dist, names(dist)
export delimited using "Subgroup_Distribution.csv", replace

* ----------------------
* 4. Primary Model: Fully Adjusted Weighted Multinomial Logistic Regression (Ref = Profile 1)
* ----------------------
mlogit LPA_Class_ID male edu_junior edu_high edu_col ///
    inc_50less inc_50_100 inc_100_150 inc_150_200 inc_200up ///
    chronic_yes businsur_yes urban i.emp_cat age ///
    [pw=Max_Posterior], baseoutcome(1)
est store full_adj

```

```
* Export primary regression table (Odds Ratios and 95% Confidence Intervals)
esttab full_adj using "Table4_Full_Adjusted_Regression.rtf", ///
    eform ci star(* 0.05 ** 0.01) replace ///
    title("Fully Adjusted Weighted Multinomial Logistic Regression") ///
    label nomtitle nonumber

* ----------------------
* 5. Regression Diagnostics
* ----------------------
* 5.1 Multicollinearity Test (Variance Inflation Factor, VIF)
reg male edu_junior edu_high edu_col ///
    inc_50less inc_50_100 inc_100_150 inc_150_200 ///
    chronic_yes businsur_yes urban i.emp_cat age
vif
estimates store vif_results
esttab vif_results using "VIF_Results.rtf", replace

* 5.2 Age linearity assumption test (quadratic term significance test)
reg male edu_junior edu_high edu_col ///
    inc_50less inc_50_100 inc_100_150 inc_150_200 ///
    chronic_yes businsur_yes urban i.emp_cat age age_sq
test age_sq
estimates store linear_test
esttab linear_test using "Age_Linear_Test.rtf", replace

* ----------------------
* 6. Sensitivity Analyses
* ----------------------
* Model 1: Categorical age groups instead of continuous age
mlogit LPA_Class_ID male edu_junior edu_high edu_col ///
    inc_50less inc_50_100 inc_100_150 inc_150_200 inc_200up ///
    chronic_yes businsur_yes urban i.emp_cat i.age_group ///
    [pw=Max_Posterior], baseoutcome(1)
est store age_cat_mod

* Model 2: Exclude occupational covariate
mlogit LPA_Class_ID male edu_junior edu_high edu_col ///
    inc_50less inc_50_100 inc_100_150 inc_150_200 inc_200up ///
    chronic_yes businsur_yes urban age ///
    [pw=Max_Posterior], baseoutcome(1)
est store no_emp_mod

* Model 3: Exclude retired participants
preserve
drop if occupation == "retired"
mlogit LPA_Class_ID male edu_junior edu_high edu_col ///
    inc_50less inc_50_100 inc_100_150 inc_150_200 inc_200up ///
    chronic_yes businsur_yes urban i.emp_cat age ///
    [pw=Max_Posterior], baseoutcome(1)
est store sens_drop_retire
restore

* Export comparison table for all sensitivity models
esttab full_adj age_cat_mod no_emp_mod sens_drop_retire ///
    using "Sensitivity_Analysis_Results.rtf", ///
    eform ci star(* 0.05 ** 0.01) replace ///
    title("Primary Model vs All Sensitivity Specifications")

* ----------------------
* 7. Age Suppression Effect Validation (Crude vs Fully Adjusted Model)
* ----------------------
mlogit LPA_Class_ID age [pw=Max_Posterior], baseoutcome(1)
est store crude_age
esttab crude_age full_adj using "Age_Suppression_Effect.rtf", ///
    eform ci star(* 0.05 ** 0.01) replace ///
    title("Crude Age Model vs Fully Adjusted Model (Suppression Effect)")


* ==============================================================================
* 8. Supplementary Robustness Checks (for Reviewer 2, Fifth Round)
* Purpose: (1) Unweighted multinomial regression (no posterior probability weights)
*          (2) Weighted regression using Class 4 as an alternative reference category
* ==============================================================================

* ------------------------------------------------------------------------------
```

```
* 8.1 Unweighted Multinomial Regression (without posterior probability weights)
* Purpose: Compare weighted vs unweighted results; assess whether weighting
*          materially affects the direction and significance of core associations
* ------------------------------------------------------------------------------
mlogit LPA_Class_ID male edu_junior edu_high edu_col ///
    inc_50less inc_50_100 inc_100_150 inc_150_200 inc_200up ///
    chronic_yes businsur_yes urban i.emp_cat age ///
    , baseoutcome(1)
est store unweighted

* ------------------------------------------------------------------------------
* 8.2 Weighted Regression Using Class 4 as Alternative Reference Category
* Purpose: Because Class 1 has a diagonal posterior probability of 0.538
*          (below the 0.70 threshold), we re-estimate the model using Class 4
*          (Moderate-Low Balanced, diagonal posterior = 0.909) as the reference
*          to verify that core associations remain stable
* ------------------------------------------------------------------------------
mlogit LPA_Class_ID male edu_junior edu_high edu_col ///
    inc_50less inc_50_100 inc_100_150 inc_150_200 inc_200up ///
    chronic_yes businsur_yes urban i.emp_cat age ///
    [pw=Max_Posterior], baseoutcome(4)
est store ref_class4

* ------------------------------------------------------------------------------
* 8.3 Export Comparison Table for Supplementary Table S9
* Purpose: Compare primary model (weighted, Class 1 ref) with the two
*          supplementary robustness checks
* ------------------------------------------------------------------------------

* Table S9a: Comparison of Weighted (Primary) vs Unweighted Models
* (Only showing Class 5 vs Class 1 for brevity, as this is the profile with the
*  strongest and most consistent associations)
esttab full_adj unweighted using "Supplementary_Table_S9a_Weighted_vs_Unweighted.rtf", ///
    eform ci star(* 0.05 ** 0.01) replace ///
    title("Supplementary Table S9a: Weighted vs Unweighted Multinomial Regression (Class 5 vs Class 1)") ///
    label nomtitle nonumber ///
    keep(male edu_junior edu_high edu_col inc_50less inc_50_100 inc_100_150 inc_150_200 chronic_yes businsur_yes urban 2.emp_cat age)

* Table S9b: Comparison of Class 1 Reference vs Class 4 Reference (Weighted Models)
esttab full_adj ref_class4 using "Supplementary_Table_S9b_Class1_vs_Class4_Reference.rtf", ///
    eform ci star(* 0.05 ** 0.01) replace ///
    title("Supplementary Table S9b: Class 1 Reference vs Class 4 Reference (Weighted Models)") ///
    label nomtitle nonumber ///
    keep(male edu_junior edu_high edu_col inc_50less inc_50_100 inc_100_150 inc_150_200 chronic_yes businsur_yes urban 2.emp_cat age)

* ------------------------------------------------------------------------------
* 8.4 Summary of Key Findings for Supplementary Table S9 (Core Associations)
* Purpose: Extract the most important ORs for Class 5 vs Class 1 across all models
*          to demonstrate stability
* ------------------------------------------------------------------------------
* Store all models for comprehensive comparison
esttab full_adj unweighted ref_class4 using "Supplementary_Table_S9_Complete.rtf", ///
    eform ci star(* 0.05 ** 0.01) replace ///
    title("Supplementary Table S9: Robustness Checks - Alternative Reference and Unweighted Models") ///
    label nomtitle nonumber ///
    keep(male edu_junior edu_high edu_col inc_50less inc_50_100 inc_100_150 inc_150_200 chronic_yes businsur_yes urban 2.emp_cat age)

* ------------------------------------------------------------------------------
* 8.5 Quick Verification: Display Key Results in Stata Output Window
* Purpose: Immediate visual confirmation that results are consistent
* ------------------------------------------------------------------------------
di as result "============================================================================"
di as result "Supplementary Robustness Checks - Key Results for Class 5 vs Class 1"
di as result "============================================================================"
di as text "Model 1 (Primary, Weighted, Ref=Class 1):"
esttab full_adj, eform ci keep(edu_junior edu_high edu_col chronic_yes businsur_yes urban
```

```
age) star(* 0.05 ** 0.01)

di as text "Model 2 (Unweighted, Ref=Class 1):"
esttab unweighted, eform ci keep(edu_junior edu_high edu_col chronic_yes businsur_yes urban age) star(* 0.05 ** 0.01)

di as text "Model 3 (Weighted, Ref=Class 4):"
esttab ref_class4, eform ci keep(edu_junior edu_high edu_col chronic_yes businsur_yes urban age) star(* 0.05 ** 0.01)
di as result "================================================================================"
di as result "Interpretation: The direction and significance of all core associations"
di as result "remain consistent across all three model specifications, confirming that"
di as result "the main findings are robust to alternative reference categories and weighting."
di as result "================================================================================"

* ==============================================================================
* End of Supplementary Robustness Checks
* ==============================================================================


* ==============================================================================
* 9. Save Stata dataset for future use
* Purpose: Export the final dataset with all variables for reproducibility
* ==============================================================================
save "LPA_Analysis_Dataset.dta", replace

* ==============================================================================
* End of do-file
* ==============================================================================
```
