# Supplementary material for: Latent profile analysis of eHealth literacy and its sociodemographic correlates: a cross-sectional study
Source: Front Public Health. 2026 Jul 27;14:1904244. doi: 10.3389/fpubh.2026.1904244 (PMC13454114; doi:10.3389/fpubh.2026.1904244)
Supplement: Supplementary file 4 [file Data_Sheet_4.PDF]

```
                                                  ___  ____  ____  ____  ____(R)
                                                 /__    /   ____/   /   ____/
                                                ___/   /   /___/   /   /___/
                                                 Statistics/Data Analysis

                                                   User: regression
-------------------------------------------------------------------------------------------------
      name:  <unnamed>
       log:  E:\ssci 教学研究论文\电子健康素养\论文 写\论文返修 0713\stata log file0713.smcl
  log type:  smcl
 opened on:  13 Jul 2026, 10:32:02

 1 . do "E:\ssci 教学研究论文\电子健康素养\论文 写\论文返修 0713\Revised stata do file 0713.do"

 2 . * ===============================================================================
 3 . * Title: Latent Profile Analysis of eHealth Literacy among Chinese Adults
 4 . * Purpose: Replicate all core regression, sensitivity and robustness analyses
 5 . * Data:    LPA_Results_With_Posteriors.xlsx (generated by R script)
 6 . * Output:  RTF tables for manuscript (Table 4, sensitivity, diagnostics, robustness)
 7 . * ===============================================================================
 8 .
 9 . clear all

10 . set more off

11 .
12 . * ----------------------
13 . * 0. Preliminary Settings
14 . * ----------------------
15 . cd "E:\ssci 教学研究论文\电子健康素养\论文 写\论文返修 0713"
   E:\ssci 教学研究论文\电子健康素养\论文 写\论文返修 0713

16 . ssc install estout, replace
   checking estout consistency and verifying not already installed...
   all files already exist and are up to date.

17 .
18 .
19 . * ----------------------
20 . * 1. Data Import & Variable Check
21 . * ----------------------
22 . import excel "LPA_Results_With_Posteriors.xlsx", firstrow clear

23 .
24 . * ----- Check existence of key variables -----
25 . capture confirm variable yingyong pingpan juece

26 . if _rc != 0 {
27 .     display as error "Key variables (yingyong, pingpan, juece) missing, please check Excel co
28 .     exit
29 . }

30 . capture confirm variable class_id

31 . if _rc != 0 {
32 .     display as error "Latent profile identifier class_id missing, verify column exists in R o
33 .     exit
34 . }

35 . capture confirm variable max_prob

36 . if _rc != 0 {
37 .     display as error "Maximum posterior probability max_prob missing, verify column exists in
38 .     exit
39 . }
```

```
40 .
41 . * ----- Standardize variable names consistent with manuscript -----
42 . rename chengxiang residence

43 . rename occupation occupation
     (all newnames==oldnames)

44 . rename age age
     (all newnames==oldnames)

45 . rename gender gender
     (all newnames==oldnames)

46 . rename education education
     (all newnames==oldnames)

47 . rename income income
     (all newnames==oldnames)

48 . rename chronic chronic
     (all newnames==oldnames)

49 . rename businsur businsur
     (all newnames==oldnames)

50 . rename yingyong application

51 . rename pingpan evaluation

52 . rename juece decision

53 .
54 . * Rename latent profile and posterior probability variables to Stata standard naming
55 . rename class_id LPA_Class_ID

56 . rename max_prob Max_Posterior

57 .
58 . * ----- Set reference group of outcome to 1 (Low eHealth Literacy) -----
59 . * Recode if class_id does not start at 1 (R output class_id ranges 1~5, no recoding needed)
60 . tab LPA_Class_ID
```

class_id	Freq.	Percent	Cum.
1	**157**	**11.29**	**11.29**
2	**413**	**29.69**	**40.98**
3	**430**	**30.91**	**71.89**
4	**254**	**18.26**	**90.15**
5	**137**	**9.85**	**100.00**
Total	**1,391**	**100.00**	

```
61 .
62 . * ---------------------
63 . * 2. Covariate Recoding & Generation
64 . * ---------------------
65 . * Occupational grouping (employed vs non-employed)
66 . gen emp_cat = .
(1,391 missing values generated)
```

```
67 . replace emp_cat = 1 if occupation == "employed"
   (1,071 real changes made)

68 . replace emp_cat = 2 if inlist(occupation, "retired", "student", "others")
   (320 real changes made)

69 . label define emp_lab 1 "Employed (reference)" 2 "Non-employed"

70 . label values emp_cat emp_lab

71 .
72 . * Age grouping for sensitivity analysis models
73 . gen age_group = 4

74 . replace age_group = 1 if age <= 34
   (666 real changes made)

75 . replace age_group = 2 if age > 34 & age <= 49
   (418 real changes made)

76 . replace age_group = 3 if age > 49 & age <= 64
   (268 real changes made)

77 . label define agelab 1 "18-34" 2 "35-49" 3 "50-64" 4 "≥65"

78 . label values age_group agelab

79 .
80 . * Quadratic age term for linearity assumption test
81 . gen age_sq = age^2

82 .
83 . * Generate dummy variables (reference categories aligned with manuscript)
84 . * Gender (female = reference 0)
85 . tab gender, gen(gender_)
```

gender	Freq.	Percent	Cum.
female	946	68.01	68.01
male	445	31.99	100.00
Total	1,391	100.00	

```
86 . drop gender_1

87 . rename gender_2 male

88 .
89 . * Education attainment (primary school and below = reference 0)
90 . tab education, gen(edu_)
```

education	Freq.	Percent	Cum.
college and above	913	65.64	65.64
high school	137	9.85	75.49
junior school	183	13.16	88.64
primary and below	158	11.36	100.00
Total	1,391	100.00	

```
 91 . rename edu_2 edu_junior

 92 . rename edu_3 edu_high

 93 . rename edu_4 edu_col

 94 . drop edu_1

 95 .
 96 . * Household income (≥200,000 CNY = reference 0)
 97 . tab income, gen(inc_)
```

income	Freq.	Percent	Cum.
100k	**246**	**17.69**	**17.69**
150k-	**107**	**7.69**	**25.38**
200k-	**171**	**12.29**	**37.67**
50k-	**364**	**26.17**	**63.84**
<50k	**503**	**36.16**	**100.00**
Total	**1,391**	**100.00**	

```
 98 . rename inc_1 inc_50less

 99 . rename inc_2 inc_50_100

100 . rename inc_3 inc_100_150

101 . rename inc_4 inc_150_200

102 . rename inc_5 inc_200up

103 .
104 .
105 . * Chronic disease status (no chronic disease = reference 0)
106 . tab chronic, gen(chro_)
```

chronic	Freq.	Percent	Cum.
no	**1,154**	**82.96**	**82.96**
yes	**237**	**17.04**	**100.00**
Total	**1,391**	**100.00**	

```
107 . drop chro_1

108 . rename chro_2 chronic_yes

109 .
110 . * Private medical insurance (no private insurance = reference 0)
111 . tab businsur, gen(ins_)
```

businsur	Freq.	Percent	Cum.
no	**1,073**	**77.14**	**77.14**
yes	**318**	**22.86**	**100.00**
Total	**1,391**	**100.00**	

```
112 . drop ins_1
```

```
. rename ins_2 businsur_yes

.
. * Residential location (rural = reference 0)
. tab residence, gen(res_)
```

chengxiang	Freq.	Percent	Cum.
rural	609	43.78	43.78
urban	782	56.22	100.00
Total	1,391	100.00	

```
. drop res_1

. rename res_2 urban

.
. * ----- Verify all dummy variables are correctly generated -----
. ds male edu_* inc_* chronic_yes businsur_yes urban
male          edu_high      inc_50less    inc_100_150   inc_200up     businsur_yes
edu_junior    edu_col       inc_50_100    inc_150_200   chronic_yes   urban

. tabstat `r(varlist)', by(LPA_Class_ID) statistics(mean)  // Quick descriptive overview

Summary statistics: mean
  by categories of: LPA_Class_ID (class_id)
```

LPA_Class_ID	male	edu_ju~r	edu_high	edu_col	inc_50~s	inc_~100	inc_~150	inc_~200
1	.3503185	.0828025	.1910828	.1656051	.1401274	.0573248	.1019108	.2993631
2	.3535109	.1138015	.1065375	.0992736	.1815981	.0750605	.1331719	.251816
3	.3023256	.0651163	.1093023	.0813953	.1837209	.0930233	.1232558	.2604651
4	.2480315	.1220472	.1102362	.0826772	.1574803	.0472441	.0866142	.2519685
5	.3722628	.1313869	.2481752	.2554745	.2189781	.1094891	.1824818	.270073
Total	.3199137	.0984903	.13156	.1135873	.1768512	.0769231	.1229331	.2616822

LPA_Class_ID	urban
1	.5796178
2	.5641646
3	.6023256
4	.5433071
5	.4452555
Total	.5621855

```
.
. * ----------------------
. * 3. Descriptive Statistics (Subscale Means & Sample Distribution)
. * ----------------------
. estpost tabstat application evaluation decision, by(LPA_Class_ID) ///
>     statistics(n mean sd) columns(statistics) listwise

Summary statistics: count mean sd
     for variables: application evaluation decision
  by categories of: LPA_Class_ID
```

LPA_Class_ID	e(count)	e(mean)	e(sd)
**1**			
application	**157**	**2.619108**	**.9037765**
evaluation	**157**	**2.340764**	**.9624991**
decision	**157**	**1.707006**	**.456592**
**2**			
application	**413**	**4.715254**	**.5982862**
evaluation	**413**	**4.801453**	**.4882581**
decision	**413**	**5**	**0**
**3**			
application	**430**	**3.950233**	**.4726925**
evaluation	**430**	**3.946512**	**.4445206**
decision	**430**	**4**	**0**
**4**			
application	**254**	**3.169291**	**.505935**
evaluation	**254**	**3.029528**	**.3977632**
decision	**254**	**3**	**0**
**5**			
application	**137**	**4.236496**	**.3844163**
evaluation	**137**	**3.985401**	**.4327647**
decision	**137**	**3**	**0**
**Total**			
application	**1391**	**3.912725**	**.9054927**
evaluation	**1391**	**3.8555**	**.9678112**
decision	**1391**	**3.757009**	**1.066113**

```
128 .
129 . esttab . using "LPA_Subgroup_Stats.csv", ///
>     cells("n mean sd") label replace nomtitle nonumber ///
>     title("Table: Subgroup Means and Standard Deviations")
(output written to LPA_Subgroup_Stats.csv)

130 .
131 . * 同时导出类别分布（频数表）
132 . tab LPA_Class_ID, matcell(subgroup_dist)
```

class_id	Freq.	Percent	Cum.
1	**157**	**11.29**	**11.29**
2	**413**	**29.69**	**40.98**
3	**430**	**30.91**	**71.89**
4	**254**	**18.26**	**90.15**
5	**137**	**9.85**	**100.00**
Total	**1,391**	**100.00**	

```
133 . svmat subgroup_dist, names(dist)

134 . export delimited using "Subgroup_Distribution.csv", replace
file Subgroup_Distribution.csv saved

135 .
136 . * ----------------------
137 . * 4. Primary Model: Fully Adjusted Weighted Multinomial Logistic Regression (Ref = Profile 1)
```

```
. * ----------------------
. mlogit LPA_Class_ID male edu_junior edu_high edu_col ///
>     inc_50less inc_50_100 inc_100_150 inc_150_200 inc_200up ///
>     chronic_yes businsur_yes urban i.emp_cat age ///
>     [pw=Max_Posterior], baseoutcome(1)

note: inc_200up omitted because of collinearity
Iteration 0:   log pseudolikelihood = -1973.8819
Iteration 1:   log pseudolikelihood = -1871.3122
Iteration 2:   log pseudolikelihood = -1847.5397
Iteration 3:   log pseudolikelihood = -1847.2765
Iteration 4:   log pseudolikelihood = -1847.2762
Iteration 5:   log pseudolikelihood = -1847.2762

Multinomial logistic regression                 Number of obs     =      1,391
                                                Wald chi2(52)     =     243.71
                                                Prob > chi2       =     0.0000
Log pseudolikelihood = -1847.2762               Pseudo R2         =     0.0641
```

LPA_Class_ID	Coef.	Robust Std. Err.	z	P>|z|	[95% Conf.	Interval]
**1**	(base outcome)					
**2**						
male	.1234276	.2024622	0.61	0.542	-.2733911	.5202462
edu_junior	.4456597	.3954463	1.13	0.260	-.3294009	1.22072
edu_high	-.4068806	.3749266	-1.09	0.278	-1.141723	.327962
edu_col	-.3163939	.3926382	-0.81	0.420	-1.085951	.4531629
inc_50less	.5148026	.303026	1.70	0.089	-.0791174	1.108723
inc_50_100	.5279339	.4264777	1.24	0.216	-.3079471	1.363815
inc_100_150	.5582388	.3549571	1.57	0.116	-.1374643	1.253942
inc_150_200	-.0211514	.2402291	-0.09	0.930	-.4919917	.449689
inc_200up	0	(omitted)				
chronic_yes	-.5863314	.2747488	-2.13	0.033	-1.124829	-.0478336
businsur_yes	.1126463	.2533002	0.44	0.657	-.3838129	.6091055
urban	-.4231473	.2199194	-1.92	0.054	-.8541815	.0078868
emp_cat						
Non-employed	.0090416	.21959	0.04	0.967	-.421347	.4394301
age	-.0228735	.0102553	-2.23	0.026	-.0429734	-.0027735
_cons	1.985329	.4014801	4.95	0.000	1.198443	2.772216
**3**						
male	-.0315732	.2041756	-0.15	0.877	-.4317499	.3686036
edu_junior	.2097475	.4071627	0.52	0.606	-.5882768	1.007772
edu_high	.1308433	.3684168	0.36	0.722	-.5912404	.852927
edu_col	.0272486	.39067	0.07	0.944	-.7384505	.7929478
inc_50less	.5088391	.304077	1.67	0.094	-.0871409	1.104819
inc_50_100	.7194935	.425431	1.69	0.091	-.1143359	1.553323
inc_100_150	.3900986	.3563878	1.09	0.274	-.3084087	1.088606
inc_150_200	.0329809	.2383619	0.14	0.890	-.4341998	.5001616
inc_200up	0	(omitted)				
chronic_yes	-.1034266	.2611957	-0.40	0.692	-.6153608	.4085076
businsur_yes	.2485908	.250553	0.99	0.321	-.2424841	.7396658
urban	-.3184473	.2159027	-1.47	0.140	-.7416089	.1047142
emp_cat						
Non-employed	-.6264975	.231857	-2.70	0.007	-1.080929	-.1720662
age	-.0489695	.0111065	-4.41	0.000	-.0707378	-.0272011
_cons	2.929779	.4154199	7.05	0.000	2.115571	3.743987
**4**						
male	-.2920795	.2266784	-1.29	0.198	-.7363611	.152202
edu_junior	.5063023	.4225765	1.20	0.231	-.3219324	1.334537
edu_high	-.3789593	.4021141	-0.94	0.346	-1.167088	.4091698
edu_col	-.618589	.4226418	-1.46	0.143	-1.446952	.2097738
inc_50less	.1077662	.3285102	0.33	0.743	-.5361019	.7516343
inc_50_100	-.1209424	.4884966	-0.25	0.804	-1.078378	.8364933
inc_100_150	-.2314794	.3919142	-0.59	0.555	-.9996171	.5366583
inc_150_200	-.3016885	.2529082	-1.19	0.233	-.7973793	.1940024

inc_200up	0	(omitted)				
chronic_yes	-.3532329	.2993058	-1.18	0.238	-.9398615	.2333958
businsur_yes	-.2096503	.2878959	-0.73	0.466	-.7739158	.3546153
urban	-.346453	.2324107	-1.49	0.136	-.8019696	.1090637
emp_cat						
Non-employed	.1328571	.2318536	0.57	0.567	-.3215676	.5872818
age	-.014976	.0113908	-1.31	0.189	-.0373016	.0073496
_cons	1.552559	.4282554	3.63	0.000	.7131937	2.391924
**5**						
male	-.0331071	.2563612	-0.13	0.897	-.5355659	.4693517
edu_junior	1.327677	.4685239	2.83	0.005	.4093865	2.245967
edu_high	1.359642	.4270074	3.18	0.001	.5227233	2.196562
edu_col	1.515375	.4512705	3.36	0.001	.6309008	2.399848
inc_50less	1.13355	.3797454	2.99	0.003	.3892627	1.877837
inc_50_100	1.128162	.5165147	2.18	0.029	.1158116	2.140512
inc_100_150	1.608672	.4356597	3.69	0.000	.7547946	2.462549
inc_150_200	.4537762	.3222928	1.41	0.159	-.1779061	1.085459
inc_200up	0	(omitted)				
chronic_yes	1.226177	.2926754	4.19	0.000	.6525439	1.79981
businsur_yes	.7226633	.2818458	2.56	0.010	.1702556	1.275071
urban	-.5458643	.2712953	-2.01	0.044	-1.077593	-.0141354
emp_cat						
Non-employed	-.3006069	.2935695	-1.02	0.306	-.8759925	.2747787
age	-.0662188	.0145584	-4.55	0.000	-.0947528	-.0376848
_cons	.9412147	.5474942	1.72	0.086	-.1318543	2.014284

```
140 . est store full_adj

141 .
142 . * Export primary regression table (Odds Ratios and 95% Confidence Intervals)
143 . esttab full_adj using "Table4_Full_Adjusted_Regression.rtf", ///
    >     eform ci star(* 0.05 ** 0.01) replace ///
    >     title("Fully Adjusted Weighted Multinomial Logistic Regression") ///
    >     label nomtitle nonumber
    (output written to Table4_Full_Adjusted_Regression.rtf)

144 .
145 . * ----------------------
146 . * 5. Regression Diagnostics
147 . * ----------------------
148 . * 5.1 Multicollinearity Test (Variance Inflation Factor, VIF)
149 . reg male edu_junior edu_high edu_col ///
    >     inc_50less inc_50_100 inc_100_150 inc_150_200 ///
    >     chronic_yes businsur_yes urban i.emp_cat age
```

Source	SS	df	MS
Model	14.1573778	12	1.17978148
Residual	288.481012	1,378	.209347614
Total	302.63839	1,390	.21772546

Number of obs	=	1,391
F(12, 1378)	=	5.64
Prob > F	=	0.0000
R-squared	=	0.0468
Adj R-squared	=	0.0385
Root MSE	=	.45755

male	Coef.	Std. Err.	t	P>|t|	[95% Conf.	Interval]
edu_junior	-.0096771	.0488509	-0.20	0.843	-.1055072	.0861531
edu_high	.0041378	.0499002	0.08	0.934	-.0937506	.1020263
edu_col	.0208906	.0541231	0.39	0.700	-.0852819	.1270631
inc_50less	.0872675	.0367056	2.38	0.018	.0152626	.1592724
inc_50_100	.0868222	.0501099	1.73	0.083	-.0114777	.1851221
inc_100_150	.0294611	.0434477	0.68	0.498	-.0557697	.1146919
inc_150_200	.0549538	.0319953	1.72	0.086	-.0078109	.1177185
chronic_yes	.0364569	.0367332	0.99	0.321	-.035602	.1085159
businsur_yes	.0013703	.0309311	0.04	0.965	-.0593069	.0620475
urban	.0157696	.0276222	0.57	0.568	-.0384165	.0699557
emp_cat						
Non-employed	-.0755095	.0302075	-2.50	0.013	-.1347671	-.0162519

age	.0060231	.0014093	4.27	0.000	.0032586	.0087877
_cons	.051738	.049547	1.04	0.297	-.0454577	.1489338

```
150 . vif
```

Variable	VIF	1/VIF
edu_junior	1.41	0.710284
edu_high	1.89	0.529022
edu_col	1.96	0.510282
inc_50less	1.30	0.767344
inc_50_100	1.18	0.844111
inc_100_150	1.35	0.739444
inc_150_200	1.31	0.760942
chronic_yes	1.27	0.789086
businsur_yes	1.12	0.892024
urban	1.25	0.801409
2.emp_cat	1.07	0.931165
age	2.24	0.445709
Mean VIF	1.45	

```
151 . estimates store vif_results

152 . esttab vif_results using "VIF_Results.rtf", replace
    (output written to VIF_Results.rtf)

153 .
154 . * 5.2 Age linearity assumption test (quadratic term significance test)
155 . reg male edu_junior edu_high edu_col ///
    >     inc_50less inc_50_100 inc_100_150 inc_150_200 ///
    >     chronic_yes businsur_yes urban i.emp_cat age age_sq
```

Source	SS	df	MS
Model	14.1810189	13	1.09084761
Residual	288.457371	1,377	.209482477
Total	302.63839	1,390	.21772546

Number of obs	=	1,391
F(13, 1377)	=	5.21
Prob > F	=	0.0000
R-squared	=	0.0469
Adj R-squared	=	0.0379
Root MSE	=	.45769

male	Coef.	Std. Err.	t	P>|t|	[95% Conf.	Interval]
edu_junior	-.0073171	.049369	-0.15	0.882	-.1041637	.0895294
edu_high	.0063972	.0503673	0.13	0.899	-.0924077	.1052021
edu_col	.0215939	.0541809	0.40	0.690	-.0846922	.12788
inc_50less	.0882136	.0368253	2.40	0.017	.0159739	.1604533
inc_50_100	.0884938	.0503724	1.76	0.079	-.0103211	.1873087
inc_100_150	.0319786	.0441031	0.73	0.469	-.0545378	.1184951
inc_150_200	.0563432	.0322717	1.75	0.081	-.0069638	.1196502
chronic_yes	.0369475	.036774	1.00	0.315	-.0351916	.1090867
businsur_yes	.002779	.031224	0.09	0.929	-.0584727	.0640307
urban	.0165771	.0277355	0.60	0.550	-.0378312	.0709855
emp_cat						
Non-employed	-.0787963	.0317617	-2.48	0.013	-.1411027	-.0164898
age	.0037697	.0068545	0.55	0.582	-.0096766	.017216
age_sq	.0000265	.000079	0.34	0.737	-.0001284	.0001815
_cons	.0928207	.1319542	0.70	0.482	-.1660323	.3516738

```
156 . test age_sq

     ( 1)  age_sq = 0

           F(  1,  1377) =    0.11
                Prob > F =    0.7370

157 . estimates store linear_test

158 . esttab linear_test using "Age_Linear_Test.rtf", replace
    (output written to Age_Linear_Test.rtf)

159 .
160 . * ---------------------
161 . * 6. Sensitivity Analyses
162 . * ---------------------
163 . * Model 1: Categorical age groups instead of continuous age
164 . mlogit LPA_Class_ID male edu_junior edu_high edu_col ///
    >     inc_50less inc_50_100 inc_100_150 inc_150_200 inc_200up ///
    >     chronic_yes businsur_yes urban i.emp_cat i.age_group ///
    >     [pw=Max_Posterior], baseoutcome(1)

    note: inc_200up omitted because of collinearity
    Iteration 0:   log pseudolikelihood = -1973.8819
    Iteration 1:   log pseudolikelihood = -1859.2021
    Iteration 2:   log pseudolikelihood = -1832.8727
    Iteration 3:   log pseudolikelihood = -1832.4409
    Iteration 4:   log pseudolikelihood = -1832.4402
    Iteration 5:   log pseudolikelihood = -1832.4402

    Multinomial logistic regression                 Number of obs     =      1,391
                                                    Wald chi2(60)     =     273.91
                                                    Prob > chi2       =     0.0000
    Log pseudolikelihood = -1832.4402               Pseudo R2         =     0.0717
```

LPA_Class_ID	Coef.	Robust Std. Err.	z	P>|z|	[95% Conf.	Interval]
**1**	(base outcome)					
**2**						
male	.0954003	.2041749	0.47	0.640	-.3047751	.4955757
edu_junior	.1747984	.3970656	0.44	0.660	-.6034358	.9530327
edu_high	-.6944574	.3809567	-1.82	0.068	-1.441119	.0522039
edu_col	-.558408	.3967957	-1.41	0.159	-1.336113	.2192973
inc_50less	.4425353	.3052893	1.45	0.147	-.1558207	1.040891
inc_50_100	.435596	.4329276	1.01	0.314	-.4129265	1.284118
inc_100_150	.3685171	.3723948	0.99	0.322	-.3613633	1.098397
inc_150_200	-.0663146	.2452232	-0.27	0.787	-.5469433	.4143141
inc_200up	0	(omitted)				
chronic_yes	-.6161351	.2778588	-2.22	0.027	-1.160728	-.0715419
businsur_yes	.0434235	.2609424	0.17	0.868	-.4680142	.5548612
urban	-.4496634	.2200797	-2.04	0.041	-.8810116	-.0183152
emp_cat						
Non-employed	.0725764	.2220439	0.33	0.744	-.3626217	.5077744
age_group						
35-49	.2386891	.2984411	0.80	0.424	-.3462446	.8236229
50-64	-.3068075	.3361939	-0.91	0.361	-.9657355	.3521206
≥65	-1.085046	.6245857	-1.74	0.082	-2.309211	.1391196
_cons	1.29439	.2410052	5.37	0.000	.8220286	1.766752
**3**						
male	-.0614897	.2045232	-0.30	0.764	-.4623478	.3393684
edu_junior	-.0169555	.4087172	-0.04	0.967	-.8180265	.7841155
edu_high	-.1397398	.3729366	-0.37	0.708	-.8706821	.5912025
edu_col	-.2363564	.3946351	-0.60	0.549	-1.009827	.5371141
inc_50less	.4860997	.3048643	1.59	0.111	-.1114233	1.083623
inc_50_100	.6876144	.4298568	1.60	0.110	-.1548894	1.530118

inc_100_150	.3269136	.3721184	0.88	0.380	-.4024251	1.056252
inc_150_200	.0199134	.2428782	0.08	0.935	-.4561192	.495946
inc_200up	0	(omitted)				
chronic_yes	-.1417186	.2632758	-0.54	0.590	-.6577297	.3742925
businsur_yes	.2059961	.257705	0.80	0.424	-.2990964	.7110885
urban	-.3219797	.2148631	-1.50	0.134	-.7431036	.0991442
emp_cat						
Non-employed	-.5700152	.2323569	-2.45	0.014	-1.025426	-.1146041
age_group						
35-49	-.5027121	.298385	-1.68	0.092	-1.087536	.0821118
50-64	-.9946875	.3507552	-2.84	0.005	-1.682155	-.3072199
≥65	-3.136003	.9993944	-3.14	0.002	-5.09478	-1.177226
_cons	1.593564	.2362153	6.75	0.000	1.13059	2.056537
**4**						
male	-.3454305	.2287555	-1.51	0.131	-.793783	.1029221
edu_junior	.4085048	.4264782	0.96	0.338	-.4273772	1.244387
edu_high	-.5217555	.4166271	-1.25	0.210	-1.33833	.2948187
edu_col	-.7847602	.4445723	-1.77	0.078	-1.656106	.0865855
inc_50less	.0783634	.3311001	0.24	0.813	-.5705809	.7273077
inc_50_100	-.1173999	.4965099	-0.24	0.813	-1.090541	.8557416
inc_100_150	-.3477855	.4091245	-0.85	0.395	-1.149655	.4540838
inc_150_200	-.2773834	.2601155	-1.07	0.286	-.7872004	.2324337
inc_200up	0	(omitted)				
chronic_yes	-.3986385	.3043746	-1.31	0.190	-.9952018	.1979248
businsur_yes	-.1976252	.2952616	-0.67	0.503	-.7763274	.381077
urban	-.3325532	.2335829	-1.42	0.155	-.7903673	.1252609
emp_cat						
Non-employed	.0913101	.2351021	0.39	0.698	-.3694815	.5521017
age_group						
35-49	.1737132	.3218554	0.54	0.589	-.4571119	.8045382
50-64	-.6589324	.3857901	-1.71	0.088	-1.415067	.0972024
≥65	.4400341	.5364458	0.82	0.412	-.6113803	1.491449
_cons	1.096584	.2472235	4.44	0.000	.6120345	1.581133
**5**						
male	-.0564599	.2575279	-0.22	0.826	-.5612052	.4482854
edu_junior	1.208549	.4697274	2.57	0.010	.2879007	2.129198
edu_high	1.231304	.4484947	2.75	0.006	.3522702	2.110337
edu_col	1.435117	.4577299	3.14	0.002	.5379827	2.332251
inc_50less	1.1251	.3834263	2.93	0.003	.3735985	1.876602
inc_50_100	1.134406	.5216512	2.17	0.030	.1119886	2.156824
inc_100_150	1.570471	.4519586	3.47	0.001	.6846485	2.456294
inc_150_200	.456431	.3284094	1.39	0.165	-.1872396	1.100102
inc_200up	0	(omitted)				
chronic_yes	1.217087	.2963474	4.11	0.000	.6362567	1.797917
businsur_yes	.6633438	.2902415	2.29	0.022	.0944808	1.232207
urban	-.5348432	.2724107	-1.96	0.050	-1.068758	-.000928
emp_cat						
Non-employed	-.2706751	.3027844	-0.89	0.371	-.8641216	.3227714
age_group						
35-49	-.8609331	.3933334	-2.19	0.029	-1.631852	-.0900139
50-64	-1.944689	.4572465	-4.25	0.000	-2.840875	-1.048502
≥65	-3.03361	.9637052	-3.15	0.002	-4.922438	-1.144783
_cons	-.8275265	.3433772	-2.41	0.016	-1.500533	-.1545196

```
165 . est store age_cat_mod

166 .
167 . * Model 2: Exclude occupational covariate
168 . mlogit LPA_Class_ID male edu_junior edu_high edu_col ///
    >     inc_50less inc_50_100 inc_100_150 inc_150_200 inc_200up ///
    >     chronic_yes businsur_yes urban age ///
    >     [pw=Max_Posterior], baseoutcome(1)

    note: inc_200up omitted because of collinearity
    Iteration 0:   log pseudolikelihood = -1973.8819
    Iteration 1:   log pseudolikelihood = -1880.4483
    Iteration 2:   log pseudolikelihood = -1856.4771
    Iteration 3:   log pseudolikelihood = -1856.1915
    Iteration 4:   log pseudolikelihood = -1856.1911
    Iteration 5:   log pseudolikelihood = -1856.1911

    Multinomial logistic regression                 Number of obs     =      1,391
                                                    Wald chi2(48)     =     231.17
                                                    Prob > chi2       =     0.0000
    Log pseudolikelihood = -1856.1911               Pseudo R2         =     0.0596
```

LPA_Class_ID	Coef.	Robust Std. Err.	z	P>|z|	[95% Conf.	Interval]
1	(base outcome)					
2						
male	.1222215	.2025983	0.60	0.546	-.2748639	.5193069
edu_junior	.4555456	.3960718	1.15	0.250	-.3207408	1.231832
edu_high	-.392264	.3731717	-1.05	0.293	-1.123667	.339139
edu_col	-.3043523	.3911262	-0.78	0.436	-1.070945	.4622409
inc_50less	.5093045	.3009235	1.69	0.091	-.0804948	1.099104
inc_50_100	.5187686	.4244156	1.22	0.222	-.3130706	1.350608
inc_100_150	.5530776	.3531208	1.57	0.117	-.1390264	1.245182
inc_150_200	-.029518	.2399522	-0.12	0.902	-.4998156	.4407796
inc_200up	0	(omitted)				
chronic_yes	-.5825667	.2737888	-2.13	0.033	-1.119183	-.0459505
businsur_yes	.1070352	.25245	0.42	0.672	-.3877578	.6018282
urban	-.4184331	.2196051	-1.91	0.057	-.8488511	.011985
age	-.0235853	.010387	-2.27	0.023	-.0439434	-.0032272
_cons	2.014008	.4005182	5.03	0.000	1.229007	2.799009
3						
male	.0011586	.2035299	0.01	0.995	-.3977526	.4000698
edu_junior	.1487291	.4083614	0.36	0.716	-.6516446	.9491028
edu_high	.0297002	.3644049	0.08	0.935	-.6845202	.7439206
edu_col	-.0323725	.3868344	-0.08	0.933	-.7905539	.7258089
inc_50less	.5664622	.3013256	1.88	0.060	-.0241252	1.157049
inc_50_100	.7809795	.4234439	1.84	0.065	-.0489553	1.610914
inc_100_150	.4669895	.35414	1.32	0.187	-.2271121	1.161091
inc_150_200	.0860691	.2371494	0.36	0.717	-.3787353	.5508734
inc_200up	0	(omitted)				
chronic_yes	-.1473132	.2613413	-0.56	0.573	-.6595328	.3649064
businsur_yes	.2736253	.2490698	1.10	0.272	-.2145425	.7617932
urban	-.2746025	.2154003	-1.27	0.202	-.6967794	.1475743
age	-.0473533	.0108909	-4.35	0.000	-.068699	-.0260076
_cons	2.696734	.4036496	6.68	0.000	1.905595	3.487872
4						
male	-.3053787	.2260345	-1.35	0.177	-.7483981	.1376407
edu_junior	.5306776	.4241214	1.25	0.211	-.300585	1.36194
edu_high	-.3491878	.3988148	-0.88	0.381	-1.130851	.4324749
edu_col	-.5931738	.4219929	-1.41	0.160	-1.420265	.2339171
inc_50less	.0859976	.3277151	0.26	0.793	-.5563123	.7283075
inc_50_100	-.1503656	.4866941	-0.31	0.757	-1.104269	.8035373
inc_100_150	-.2579413	.3914994	-0.66	0.510	-1.025266	.5093834
inc_150_200	-.3258044	.2537465	-1.28	0.199	-.8231385	.1715297
inc_200up	0	(omitted)				
chronic_yes	-.3464684	.2970441	-1.17	0.243	-.9286641	.2357274
businsur_yes	-.2266079	.2871254	-0.79	0.430	-.7893634	.3361476

urban	-.3554705	.232056	-1.53	0.126	-.8102919	.0993509
age	-.0158077	.0116588	-1.36	0.175	-.0386586	.0070432
_cons	1.637897	.4273499	3.83	0.000	.8003064	2.475487
**5**						
male	-.0164218	.2564497	-0.06	0.949	-.5190538	.4862103
edu_junior	1.313196	.4684667	2.80	0.005	.3950182	2.231374
edu_high	1.327711	.4244856	3.13	0.002	.4957345	2.159687
edu_col	1.504897	.4490738	3.35	0.001	.6247284	2.385065
inc_50less	1.159842	.3786111	3.06	0.002	.4177782	1.901906
inc_50_100	1.150355	.5189348	2.22	0.027	.1332615	2.167449
inc_100_150	1.645297	.433076	3.80	0.000	.7964836	2.49411
inc_150_200	.4693202	.3215158	1.46	0.144	-.1608392	1.09948
inc_200up	0	(omitted)				
chronic_yes	1.202948	.2915134	4.13	0.000	.6315919	1.774303
businsur_yes	.732182	.2807717	2.61	0.009	.1818797	1.282484
urban	-.5210096	.2697666	-1.93	0.053	-1.049742	.0077233
age	-.0665943	.0144528	-4.61	0.000	-.0949214	-.0382673
_cons	.8619243	.5336908	1.62	0.106	-.1840905	1.907939

```
169 . est store no_emp_mod

170 .
171 . * Model 3: Exclude retired participants
172 . preserve

173 . drop if occupation == "retired"
    (34 observations deleted)

174 . mlogit LPA_Class_ID male edu_junior edu_high edu_col ///
    >     inc_50less inc_50_100 inc_100_150 inc_150_200 inc_200up ///
    >     chronic_yes businsur_yes urban i.emp_cat age ///
    >     [pw=Max_Posterior], baseoutcome(1)

    note: inc_200up omitted because of collinearity
    Iteration 0:   log pseudolikelihood = -1914.0038
    Iteration 1:   log pseudolikelihood = -1823.0952
    Iteration 2:   log pseudolikelihood = -1795.6109
    Iteration 3:   log pseudolikelihood =  -1795.159
    Iteration 4:   log pseudolikelihood = -1795.1585
    Iteration 5:   log pseudolikelihood = -1795.1585

    Multinomial logistic regression                 Number of obs     =      1,357
                                                    Wald chi2(52)     =     234.54
                                                    Prob > chi2       =     0.0000
    Log pseudolikelihood = -1795.1585               Pseudo R2         =     0.0621
```

LPA_Class_ID	Coef.	Robust Std. Err.	z	P>|z|	[95% Conf.	Interval]
**1**	(base outcome)					
**2**						
male	.1028143	.2088798	0.49	0.623	-.3065825	.5122112
edu_junior	.3549419	.4154682	0.85	0.393	-.4593609	1.169245
edu_high	-.5011757	.4133322	-1.21	0.225	-1.311292	.3089404
edu_col	-.2374446	.429593	-0.55	0.580	-1.079431	.6045422
inc_50less	.3930402	.3115394	1.26	0.207	-.2175658	1.003646
inc_50_100	.57453	.4476694	1.28	0.199	-.302886	1.451946
inc_100_150	.565354	.3674989	1.54	0.124	-.1549307	1.285639
inc_150_200	-.0173964	.2525087	-0.07	0.945	-.5123043	.4775115
inc_200up	0	(omitted)				
chronic_yes	-.4103488	.308115	-1.33	0.183	-1.014243	.1935454
businsur_yes	.3395134	.2790843	1.22	0.224	-.2074818	.8865086
urban	-.4766227	.2315554	-2.06	0.040	-.9304629	-.0227824
emp_cat						
Non-employed	.3027449	.2595222	1.17	0.243	-.2059092	.811399
age	-.0186846	.0114404	-1.63	0.102	-.0411074	.0037381
_cons	1.805541	.4355544	4.15	0.000	.9518697	2.659211

**3**						
male	**-.0716099**	**.2094245**	**-0.34**	**0.732**	**-.4820744**	**.3388545**
edu_junior	**.0408793**	**.4255184**	**0.10**	**0.923**	**-.7931214**	**.87488**
edu_high	**.0561514**	**.3980625**	**0.14**	**0.888**	**-.7240367**	**.8363396**
edu_col	**.0107695**	**.4273573**	**0.03**	**0.980**	**-.8268354**	**.8483745**
inc_50less	**.3986367**	**.3098265**	**1.29**	**0.198**	**-.208612**	**1.005885**
inc_50_100	**.7443091**	**.44445**	**1.67**	**0.094**	**-.1267969**	**1.615415**
inc_100_150	**.3607529**	**.3670884**	**0.98**	**0.326**	**-.3587271**	**1.080233**
inc_150_200	**.0278285**	**.2487433**	**0.11**	**0.911**	**-.4596994**	**.5153565**
inc_200up	**0**	(omitted)				
chronic_yes	**.0738911**	**.2908035**	**0.25**	**0.799**	**-.4960732**	**.6438554**
businsur_yes	**.449698**	**.2753711**	**1.63**	**0.102**	**-.0900195**	**.9894155**
urban	**-.3515402**	**.226586**	**-1.55**	**0.121**	**-.7956405**	**.0925601**
emp_cat						
Non-employed	**-.2731276**	**.268703**	**-1.02**	**0.309**	**-.7997758**	**.2535207**
age	**-.0413982**	**.0121206**	**-3.42**	**0.001**	**-.0651541**	**-.0176423**
_cons	**2.64845**	**.4468986**	**5.93**	**0.000**	**1.772545**	**3.524355**
**4**						
male	**-.3818584**	**.234514**	**-1.63**	**0.103**	**-.8414973**	**.0777805**
edu_junior	**.3002358**	**.4502836**	**0.67**	**0.505**	**-.5823038**	**1.182775**
edu_high	**-.4038068**	**.4363063**	**-0.93**	**0.355**	**-1.258951**	**.4513379**
edu_col	**-.6650598**	**.4608712**	**-1.44**	**0.149**	**-1.568351**	**.2382312**
inc_50less	**.050352**	**.33592**	**0.15**	**0.881**	**-.608039**	**.7087431**
inc_50_100	**-.0835897**	**.5088112**	**-0.16**	**0.870**	**-1.080841**	**.913662**
inc_100_150	**-.2824984**	**.4055823**	**-0.70**	**0.486**	**-1.077425**	**.5124283**
inc_150_200	**-.3233045**	**.2658077**	**-1.22**	**0.224**	**-.844278**	**.1976691**
inc_200up	**0**	(omitted)				
chronic_yes	**-.2237189**	**.3338676**	**-0.67**	**0.503**	**-.8780875**	**.4306496**
businsur_yes	**.0282828**	**.3099131**	**0.09**	**0.927**	**-.5791356**	**.6357013**
urban	**-.4027195**	**.2449059**	**-1.64**	**0.100**	**-.8827262**	**.0772872**
emp_cat						
Non-employed	**.4655913**	**.2709588**	**1.72**	**0.086**	**-.0654783**	**.9966608**
age	**-.0072564**	**.0125528**	**-0.58**	**0.563**	**-.0318595**	**.0173467**
_cons	**1.28768**	**.4640637**	**2.77**	**0.006**	**.3781321**	**2.197228**
**5**						
male	**-.0645352**	**.2646051**	**-0.24**	**0.807**	**-.5831517**	**.4540814**
edu_junior	**1.191892**	**.4814833**	**2.48**	**0.013**	**.2482023**	**2.135582**
edu_high	**1.262722**	**.4501243**	**2.81**	**0.005**	**.3804942**	**2.144949**
edu_col	**1.471407**	**.4812599**	**3.06**	**0.002**	**.5281554**	**2.41466**
inc_50less	**1.025188**	**.3918031**	**2.62**	**0.009**	**.2572681**	**1.793108**
inc_50_100	**1.1605**	**.5403312**	**2.15**	**0.032**	**.10147**	**2.219529**
inc_100_150	**1.534314**	**.4518883**	**3.40**	**0.001**	**.6486295**	**2.419999**
inc_150_200	**.4459417**	**.3338785**	**1.34**	**0.182**	**-.2084481**	**1.100332**
inc_200up	**0**	(omitted)				
chronic_yes	**1.400339**	**.318205**	**4.40**	**0.000**	**.7766688**	**2.024009**
businsur_yes	**.9394395**	**.3054654**	**3.08**	**0.002**	**.3407382**	**1.538141**
urban	**-.6086082**	**.283001**	**-2.15**	**0.032**	**-1.16328**	**-.0539365**
emp_cat						
Non-employed	**.0844984**	**.3260305**	**0.26**	**0.796**	**-.5545097**	**.7235065**
age	**-.0576323**	**.0151809**	**-3.80**	**0.000**	**-.0873863**	**-.0278783**
_cons	**.6264552**	**.5736941**	**1.09**	**0.275**	**-.4979646**	**1.750875**

```
175 . est store sens_drop_retire
```

```
176 . restore

177 .
178 . * Export comparison table for all sensitivity models
179 . esttab full_adj age_cat_mod no_emp_mod sens_drop_retire ///
    >     using "Sensitivity_Analysis_Results.rtf", ///
    >     eform ci star(* 0.05 ** 0.01) replace ///
    >     title("Primary Model vs All Sensitivity Specifications")
    (output written to Sensitivity_Analysis_Results.rtf)

180 .
181 . * ----------------------
182 . * 7. Age Suppression Effect Validation (Crude vs Fully Adjusted Model)
183 . * ----------------------
184 . mlogit LPA_Class_ID age [pw=Max_Posterior], baseoutcome(1)

    Iteration 0:   log pseudolikelihood = -1973.8819
    Iteration 1:   log pseudolikelihood = -1944.7188
    Iteration 2:   log pseudolikelihood = -1944.1891
    Iteration 3:   log pseudolikelihood =  -1944.189

    Multinomial logistic regression               Number of obs     =      1,391
                                                  Wald chi2(4)      =      66.15
                                                  Prob > chi2       =     0.0000
    Log pseudolikelihood =  -1944.189             Pseudo R2         =     0.0150
```

LPA_Class_ID		Coef.	Robust Std. Err.	z	P>|z|	[95% Conf.	Interval]
1		(base outcome)					
2							
	age	-.0301296	.0075015	-4.02	0.000	-.0448322	-.015427
	_cons	2.136006	.3232168	6.61	0.000	1.502513	2.769499
3							
	age	-.0449587	.0076357	-5.89	0.000	-.0599244	-.0299931
	_cons	2.720017	.3234648	8.41	0.000	2.086038	3.353997
4							
	age	-.0288298	.008791	-3.28	0.001	-.0460599	-.0115997
	_cons	1.589842	.3672839	4.33	0.000	.8699792	2.309706
5							
	age	.002631	.0082612	0.32	0.750	-.0135607	.0188227
	_cons	-.2555062	.3687267	-0.69	0.488	-.9781972	.4671848

```
185 . est store crude_age

186 . esttab crude_age full_adj using "Age_Suppression_Effect.rtf", ///
    >     eform ci star(* 0.05 ** 0.01) replace ///
    >     title("Crude Age Model vs Fully Adjusted Model (Suppression Effect)")
    (output written to Age_Suppression_Effect.rtf)

187 .
188 .
189 . * ========================================================================
190 . * 8. Supplementary Robustness Checks (for Reviewer 2, Fifth Round)
```

```
. * Purpose: (1) Unweighted multinomial regression (no posterior probability weights)
. *          (2) Weighted regression using Class 4 as an alternative reference category
. * ===============================================================================
.
. * -------------------------------------------------------------------------------
. * 8.1 Unweighted Multinomial Regression (without posterior probability weights)
. * Purpose: Compare weighted vs unweighted results; assess whether weighting
. *          materially affects the direction and significance of core associations
. * -------------------------------------------------------------------------------
. mlogit LPA_Class_ID male edu_junior edu_high edu_col ///
>     inc_50less inc_50_100 inc_100_150 inc_150_200 inc_200up ///
>     chronic_yes businsur_yes urban i.emp_cat age ///
>     , baseoutcome(1)

note: inc_200up omitted because of collinearity
Iteration 0:   log likelihood = -2098.2871
Iteration 1:   log likelihood = -1997.1983
Iteration 2:   log likelihood = -1973.9165
Iteration 3:   log likelihood = -1973.6879
Iteration 4:   log likelihood = -1973.6876
Iteration 5:   log likelihood = -1973.6876

Multinomial logistic regression                 Number of obs     =      1,391
                                                LR chi2(52)       =     249.20
                                                Prob > chi2       =     0.0000
Log likelihood = -1973.6876                     Pseudo R2         =     0.0594
```

LPA_Class_ID	Coef.	Std. Err.	z	P>|z|	[95% Conf.	Interval]
**1**	(base outcome)					
**2**						
male	.1440867	.2039013	0.71	0.480	-.2555525	.5437259
edu_junior	.4895207	.3870044	1.26	0.206	-.268994	1.248035
edu_high	-.3835861	.3598091	-1.07	0.286	-1.088799	.3216268
edu_col	-.2841949	.3890606	-0.73	0.465	-1.04674	.4783499
inc_50less	.4710918	.2962066	1.59	0.112	-.1094626	1.051646
inc_50_100	.487044	.4201035	1.16	0.246	-.3363437	1.310432
inc_100_150	.4654139	.3463649	1.34	0.179	-.2134489	1.144277
inc_150_200	-.0430012	.2380473	-0.18	0.857	-.5095654	.4235629
inc_200up	0	(omitted)				
chronic_yes	-.5262978	.2732936	-1.93	0.054	-1.061943	.0093478
businsur_yes	.0701669	.2420914	0.29	0.772	-.4043235	.5446573
urban	-.4399892	.2164192	-2.03	0.042	-.864163	-.0158155
emp_cat						
Non-employed	.001792	.2254689	0.01	0.994	-.4401188	.4437029
age	-.0184111	.0102315	-1.80	0.072	-.0384644	.0016423
_cons	1.86706	.3866409	4.83	0.000	1.109258	2.624862
**3**						
male	-.0698797	.2060723	-0.34	0.735	-.473774	.3340146
edu_junior	.1534371	.408607	0.38	0.707	-.6474179	.9542922
edu_high	.0647294	.3635298	0.18	0.859	-.6477758	.7772347
edu_col	-.0279086	.4000575	-0.07	0.944	-.812007	.7561897
inc_50less	.5245533	.2970183	1.77	0.077	-.0575918	1.106699
inc_50_100	.7284019	.4130614	1.76	0.078	-.0811835	1.537987
inc_100_150	.3968985	.3487339	1.14	0.255	-.2866074	1.080404
inc_150_200	.0166623	.2383503	0.07	0.944	-.4504957	.4838203
inc_200up	0	(omitted)				
chronic_yes	-.1523419	.2724069	-0.56	0.576	-.6862496	.3815657
businsur_yes	.2240871	.2402911	0.93	0.351	-.2468748	.6950491
urban	-.3504765	.2168337	-1.62	0.106	-.7754627	.0745098
emp_cat						
Non-employed	-.5553268	.235935	-2.35	0.019	-1.017751	-.0929027
age	-.043569	.0108023	-4.03	0.000	-.0647412	-.0223969
_cons	2.793648	.396352	7.05	0.000	2.016812	3.570483
**4**						
male	-.3263224	.2290053	-1.42	0.154	-.7751646	.1225198

```
     edu_junior |   .4897266   .4098695     1.19   0.232    -.3136028    1.293056
       edu_high |  -.4963649   .3944542    -1.26   0.208    -1.269481    .2767511
        edu_col |  -.6301447   .4376343    -1.44   0.150    -1.487892    .2276028
     inc_50less |   .1448208   .3203319     0.45   0.651    -.4830182    .7726599
     inc_50_100 |  -.1810039    .480505    -0.38   0.706    -1.122776    .7607686
    inc_100_150 |  -.1828111   .3902886    -0.47   0.639    -.9477626    .5821405
    inc_150_200 |  -.2598854   .2551869    -1.02   0.308    -.7600426    .2402717
      inc_200up |          0  (omitted)
    chronic_yes |  -.2905945   .2982869    -0.97   0.330    -.8752261    .2940372
   businsur_yes |  -.2168593   .2738458    -0.79   0.428    -.7535873    .3198687
          urban |  -.3581083   .2318166    -1.54   0.122    -.8124606     .096244
                |
        emp_cat |
Non-employed    |   .1468873   .2389994     0.61   0.539     -.321543    .6153176
            age |  -.0128414    .011058    -1.16   0.246    -.0345147    .0088318
          _cons |   1.514738   .4105778     3.69   0.000     .7100206    2.319456
----------------+----------------------------------------------------------------
5               |
           male |  -.0406422   .2607806    -0.16   0.876    -.5517629    .4704784
     edu_junior |   1.248861   .4992153     2.50   0.012     .2704171    2.227305
       edu_high |   1.256736   .4651741     2.70   0.007      .345011     2.16846
        edu_col |   1.425527   .4948239     2.88   0.004     .4556901    2.395364
     inc_50less |   1.156665   .3769725     3.07   0.002     .4178126    1.895517
     inc_50_100 |    1.18408   .5053658     2.34   0.019     .1935813    2.174579
    inc_100_150 |   1.614231   .4292382     3.76   0.000     .7729397    2.455523
    inc_150_200 |   .4968619   .3250636     1.53   0.126     -.140251    1.133975
      inc_200up |          0  (omitted)
    chronic_yes |   1.211536   .3067197     3.95   0.000     .6103768    1.812696
   businsur_yes |   .7114726   .2823674     2.52   0.012     .1580427    1.264903
          urban |  -.5467525    .278002    -1.97   0.049    -1.091626   -.0018785
                |
        emp_cat |
Non-employed    |  -.2362288   .2930523    -0.81   0.420    -.8106007    .3381431
            age |  -.0627924   .0152233    -4.12   0.000    -.0926295   -.0329553
          _cons |   .8430502   .5551619     1.52   0.129    -.2450471    1.931148
---------------------------------------------------------------------------------

201 . est store unweighted

202 .
203 . * --------------------------------------------------------------------------------
204 . * 8.2 Weighted Regression Using Class 4 as Alternative Reference Category
205 . * Purpose: Because Class 1 has a diagonal posterior probability of 0.538
206 . *          (below the 0.70 threshold), we re-estimate the model using Class 4
207 . *          (Moderate-Low Balanced, diagonal posterior = 0.909) as the reference
208 . *          to verify that core associations remain stable
209 . * --------------------------------------------------------------------------------
210 . mlogit LPA_Class_ID male edu_junior edu_high edu_col ///
>     inc_50less inc_50_100 inc_100_150 inc_150_200 inc_200up ///
>     chronic_yes businsur_yes urban i.emp_cat age ///
>     [pw=Max_Posterior], baseoutcome(4)

note: inc_200up omitted because of collinearity
Iteration 0:   log pseudolikelihood = -1973.8819
Iteration 1:   log pseudolikelihood = -1871.3122
Iteration 2:   log pseudolikelihood = -1847.5397
Iteration 3:   log pseudolikelihood = -1847.2765
Iteration 4:   log pseudolikelihood = -1847.2762
Iteration 5:   log pseudolikelihood = -1847.2762

Multinomial logistic regression                 Number of obs     =      1,391
                                                Wald chi2(52)     =     243.71
                                                Prob > chi2       =     0.0000
Log pseudolikelihood = -1847.2762               Pseudo R2         =     0.0641
```

LPA_Class_ID	Coef.	Robust Std. Err.	z	P>|z|	[95% Conf.	Interval]
**1**						
male	.2920795	.2266784	1.29	0.198	-.152202	.7363611
edu_junior	-.5063023	.4225765	-1.20	0.231	-1.334537	.3219324
edu_high	.3789593	.4021141	0.94	0.346	-.4091698	1.167088
edu_col	.618589	.4226418	1.46	0.143	-.2097738	1.446952
inc_50less	-.1077662	.3285102	-0.33	0.743	-.7516343	.5361019
inc_50_100	.1209424	.4884966	0.25	0.804	-.8364933	1.078378
inc_100_150	.2314794	.3919142	0.59	0.555	-.5366583	.9996171
inc_150_200	.3016885	.2529082	1.19	0.233	-.1940024	.7973793
inc_200up	0	(omitted)				
chronic_yes	.3532329	.2993058	1.18	0.238	-.2333958	.9398615
businsur_yes	.2096503	.2878959	0.73	0.466	-.3546153	.7739158
urban	.346453	.2324107	1.49	0.136	-.1090637	.8019696
emp_cat						
Non-employed	-.1328571	.2318536	-0.57	0.567	-.5872818	.3215676
age	.014976	.0113908	1.31	0.189	-.0073496	.0373016
_cons	-1.552559	.4282554	-3.63	0.000	-2.391924	-.7131937
**2**						
male	.4155071	.1859229	2.23	0.025	.0511049	.7799093
edu_junior	-.0606426	.3124606	-0.19	0.846	-.6730542	.5517689
edu_high	-.0279214	.35545	-0.08	0.937	-.7245906	.6687479
edu_col	.3021951	.3725767	0.81	0.417	-.4280418	1.032432
inc_50less	.4070364	.2446838	1.66	0.096	-.0725351	.886608
inc_50_100	.6488763	.3761997	1.72	0.085	-.0884616	1.386214
inc_100_150	.7897182	.2985294	2.65	0.008	.2046114	1.374825
inc_150_200	.2805371	.2093369	1.34	0.180	-.1297556	.6908298
inc_200up	0	(omitted)				
chronic_yes	-.2330985	.2839397	-0.82	0.412	-.7896101	.3234131
businsur_yes	.3222965	.2301878	1.40	0.161	-.1288632	.7734563
urban	-.0766943	.1830567	-0.42	0.675	-.4354789	.2820902
emp_cat						
Non-employed	-.1238156	.1886084	-0.66	0.512	-.4934813	.2458502
age	-.0078975	.0091387	-0.86	0.387	-.025809	.0100139
_cons	.4327704	.3129661	1.38	0.167	-.1806319	1.046173
**3**						
male	.2605064	.1873603	1.39	0.164	-.1067131	.6277258
edu_junior	-.2965548	.3299272	-0.90	0.369	-.9432004	.3500907
edu_high	.5098025	.3560155	1.43	0.152	-.187975	1.20758
edu_col	.6458376	.3743375	1.73	0.084	-.0878504	1.379526
inc_50less	.4010729	.2469855	1.62	0.104	-.0830099	.8851557
inc_50_100	.8404359	.3752908	2.24	0.025	.1048793	1.575992
inc_100_150	.6215781	.3024841	2.05	0.040	.0287201	1.214436
inc_150_200	.3346693	.2081486	1.61	0.108	-.0732944	.742633
inc_200up	0	(omitted)				
chronic_yes	.2498062	.2734723	0.91	0.361	-.2861896	.7858021
businsur_yes	.4582411	.2290308	2.00	0.045	.0093489	.9071333
urban	.0280056	.1810063	0.15	0.877	-.3267602	.3827715
emp_cat						
Non-employed	-.7593546	.2011086	-3.78	0.000	-1.15352	-.3651889
age	-.0339935	.0099773	-3.41	0.001	-.0535487	-.0144384
_cons	1.377221	.3262903	4.22	0.000	.7377032	2.016738
**4**	(base outcome)					
**5**						
male	.2589725	.2466038	1.05	0.294	-.224362	.742307
edu_junior	.8213742	.4038262	2.03	0.042	.0298894	1.612859
edu_high	1.738602	.4152752	4.19	0.000	.9246773	2.552526
edu_col	2.133964	.4394748	4.86	0.000	1.272609	2.995318
inc_50less	1.025784	.339235	3.02	0.002	.3608954	1.690672
inc_50_100	1.249104	.4835869	2.58	0.010	.3012914	2.196917
inc_100_150	1.840151	.3955118	4.65	0.000	1.064963	2.61534
inc_150_200	.7554647	.306139	2.47	0.014	.1554433	1.355486

```
     inc_200up |          0  (omitted)
   chronic_yes |    1.57941   .3015932     5.24   0.000     .9882982    2.170522
  businsur_yes |   .9323135   .2642002     3.53   0.000     .4144907    1.450136
         urban |  -.1994114   .2508561    -0.79   0.427    -.6910803    .2922576
               |
       emp_cat |
Non-employed   |  -.4334641    .268216    -1.62   0.106    -.9591578    .0922297
           age |  -.0512429   .0136922    -3.74   0.000    -.0780791   -.0244066
         _cons |  -.6113442   .4833792    -1.26   0.206     -1.55875    .3360616
-------------------------------------------------------------------------------

. est store ref_class4

. 
. * ---------------------------------------------------------------------------
. * 8.3 Export Comparison Table for Supplementary Table S9
. * Purpose: Compare primary model (weighted, Class 1 ref) with the two
. *          supplementary robustness checks
. * ---------------------------------------------------------------------------
. 
. * Table S9a: Comparison of Weighted (Primary) vs Unweighted Models
. * (Only showing Class 5 vs Class 1 for brevity, as this is the profile with the
. *  strongest and most consistent associations)
. esttab full_adj unweighted using "Supplementary_Table_S9a_Weighted_vs_Unweighted.rtf", ///
>     eform ci star(* 0.05 ** 0.01) replace ///
>     title("Supplementary Table S9a: Weighted vs Unweighted Multinomial Regression (Class 5 vs
>     label nomtitle nonumber ///
>     keep(male edu_junior edu_high edu_col inc_50less inc_50_100 inc_100_150 inc_150_200 chron
(output written to Supplementary_Table_S9a_Weighted_vs_Unweighted.rtf)

. 
. * Table S9b: Comparison of Class 1 Reference vs Class 4 Reference (Weighted Models)
. esttab full_adj ref_class4 using "Supplementary_Table_S9b_Class1_vs_Class4_Reference.rtf", //
>     eform ci star(* 0.05 ** 0.01) replace ///
>     title("Supplementary Table S9b: Class 1 Reference vs Class 4 Reference (Weighted Models)"
>     label nomtitle nonumber ///
>     keep(male edu_junior edu_high edu_col inc_50less inc_50_100 inc_100_150 inc_150_200 chron
(output written to Supplementary_Table_S9b_Class1_vs_Class4_Reference.rtf)

. 
. * ---------------------------------------------------------------------------
. * 8.4 Summary of Key Findings for Supplementary Table S9 (Core Associations)
. * Purpose: Extract the most important ORs for Class 5 vs Class 1 across all models
. *          to demonstrate stability
. * ---------------------------------------------------------------------------
. * Store all models for comprehensive comparison
. esttab full_adj unweighted ref_class4 using "Supplementary_Table_S9_Complete.rtf", ///
>     eform ci star(* 0.05 ** 0.01) replace ///
>     title("Supplementary Table S9: Robustness Checks - Alternative Reference and Unweighted M
>     label nomtitle nonumber ///
>     keep(male edu_junior edu_high edu_col inc_50less inc_50_100 inc_100_150 inc_150_200 chron
(output written to Supplementary_Table_S9_Complete.rtf)

. 
. * ---------------------------------------------------------------------------
. * 8.5 Quick Verification: Display Key Results in Stata Output Window
. * Purpose: Immediate visual confirmation that results are consistent
. * ---------------------------------------------------------------------------
. di as result "==================================================================================
================================================================================
```

```
240 . di as result "Supplementary Robustness Checks - Key Results for Class 5 vs Class 1"
    Supplementary Robustness Checks - Key Results for Class 5 vs Class 1

241 . di as result "==========================================================================
    ================================================================================

242 . di as text "Model 1 (Primary, Weighted, Ref=Class 1):"
    Model 1 (Primary, Weighted, Ref=Class 1):

243 . esttab full_adj, eform ci keep(edu_junior edu_high edu_col chronic_yes businsur_yes urban age
```

	(1) LPA_Class_ID
**1**	
edu_junior	**1**
	[**1**,**1**]
edu_high	**1**
	[**1**,**1**]
edu_col	**1**
	[**1**,**1**]
chronic_yes	**1**
	[**1**,**1**]
businsur_yes	**1**
	[**1**,**1**]
urban	**1**
	[**1**,**1**]
age	**1**
	[**1**,**1**]
**2**	
edu_junior	**1.562**
	[**0.719**,**3.390**]
edu_high	**0.666**
	[**0.319**,**1.388**]
edu_col	**0.729**
	[**0.338**,**1.573**]
chronic_yes	**0.556***
	[**0.325**,**0.953**]
businsur_yes	**1.119**
	[**0.681**,**1.839**]
urban	**0.655**
	[**0.426**,**1.008**]
age	**0.977***
	[**0.958**,**0.997**]
**3**	
edu_junior	**1.233**
	[**0.555**,**2.739**]
edu_high	**1.140**
	[**0.554**,**2.347**]
edu_col	**1.028**
	[**0.478**,**2.210**]
chronic_yes	**0.902**
	[**0.540**,**1.505**]

businsur_yes	**1.282**
	[**0.785,2.095**]
urban	**0.727**
	[**0.476,1.110**]
age	**0.952****
	[**0.932,0.973**]
**4**	
edu_junior	**1.659**
	[**0.725,3.798**]
edu_high	**0.685**
	[**0.311,1.506**]
edu_col	**0.539**
	[**0.235,1.233**]
chronic_yes	**0.702**
	[**0.391,1.263**]
businsur_yes	**0.811**
	[**0.461,1.426**]
urban	**0.707**
	[**0.448,1.115**]
age	**0.985**
	[**0.963,1.007**]
**5**	
edu_junior	**3.772****
	[**1.506,9.450**]
edu_high	**3.895****
	[**1.687,8.994**]
edu_col	**4.551****
	[**1.879,11.02**]
chronic_yes	**3.408****
	[**1.920,6.048**]
businsur_yes	**2.060***
	[**1.186,3.579**]
urban	**0.579***
	[**0.340,0.986**]
age	**0.936****
	[**0.910,0.963**]
N	**1391**

Exponentiated coefficients; 95% confidence intervals in brackets
* p<0.05, ** p<0.01

```
244 .
245 . di as text "Model 2 (Unweighted, Ref=Class 1):"
    Model 2 (Unweighted, Ref=Class 1):
```

```
246 . esttab unweighted, eform ci keep(edu_junior edu_high edu_col chronic_yes businsur_yes urban a

------------------------------------------------
                                 (1)
                        LPA_Class_ID
------------------------------------------------
1
edu_junior                        1
                              [1,1]

edu_high                          1
                              [1,1]

edu_col                           1
                              [1,1]

chronic_yes                       1
                              [1,1]

businsur_yes                      1
                              [1,1]

urban                             1
                              [1,1]

age                               1
                              [1,1]
------------------------------------------------
2
edu_junior                    1.632
                      [0.764,3.483]

edu_high                      0.681
                      [0.337,1.379]

edu_col                       0.753
                      [0.351,1.613]

chronic_yes                   0.591
                      [0.346,1.009]

businsur_yes                  1.073
                      [0.667,1.724]

urban                         0.644*
                      [0.421,0.984]

age                           0.982
                      [0.962,1.002]
------------------------------------------------
3
edu_junior                    1.166
                      [0.523,2.597]

edu_high                      1.067
                      [0.523,2.175]

edu_col                       0.972
                      [0.444,2.130]

chronic_yes                   0.859
                      [0.503,1.465]

businsur_yes                  1.251
                      [0.781,2.004]

urban                         0.704
                      [0.460,1.077]
```

```
age                             0.957**
                          [0.937,0.978]
-----------------------------------------
4
edu_junior                      1.632
                          [0.731,3.644]

edu_high                        0.609
                          [0.281,1.319]

edu_col                         0.533
                          [0.226,1.256]

chronic_yes                     0.748
                          [0.417,1.342]

businsur_yes                    0.805
                          [0.471,1.377]

urban                           0.699
                          [0.444,1.101]

age                             0.987
                          [0.966,1.009]
-----------------------------------------
5
edu_junior                      3.486*
                          [1.311,9.275]

edu_high                        3.514**
                          [1.412,8.745]

edu_col                         4.160**
                          [1.577,10.97]

chronic_yes                     3.359**
                          [1.841,6.127]

businsur_yes                    2.037*
                          [1.171,3.543]

urban                           0.579*
                          [0.336,0.998]

age                             0.939**
                          [0.912,0.968]
-----------------------------------------
N                                1391
-----------------------------------------
Exponentiated coefficients; 95% confidence intervals in brackets
* p<0.05, ** p<0.01

247 .
248 . di as text "Model 3 (Weighted, Ref=Class 4):"
    Model 3 (Weighted, Ref=Class 4):

249 . esttab ref_class4, eform ci keep(edu_junior edu_high edu_col chronic_yes businsur_yes urban a

-----------------------------------------
                                  (1)
                           LPA_Class_ID
-----------------------------------------
1
edu_junior                      0.603
                          [0.263,1.380]

edu_high                        1.461
                          [0.664,3.213]

edu_col                         1.856
                          [0.811,4.250]
```

chronic_yes	**1.424**
	[**0.792,2.560**]
businsur_yes	**1.233**
	[**0.701,2.168**]
urban	**1.414**
	[**0.897,2.230**]
age	**1.015**
	[**0.993,1.038**]
**2**	
edu_junior	**0.941**
	[**0.510,1.736**]
edu_high	**0.972**
	[**0.485,1.952**]
edu_col	**1.353**
	[**0.652,2.808**]
chronic_yes	**0.792**
	[**0.454,1.382**]
businsur_yes	**1.380**
	[**0.879,2.167**]
urban	**0.926**
	[**0.647,1.326**]
age	**0.992**
	[**0.975,1.010**]
**3**	
edu_junior	**0.743**
	[**0.389,1.419**]
edu_high	**1.665**
	[**0.829,3.345**]
edu_col	**1.908**
	[**0.916,3.973**]
chronic_yes	**1.284**
	[**0.751,2.194**]
businsur_yes	**1.581*****
	[**1.009,2.477**]
urban	**1.028**
	[**0.721,1.466**]
age	**0.967******
	[**0.948,0.986**]
**4**	
edu_junior	**1**
	[**1,1**]
edu_high	**1**
	[**1,1**]
edu_col	**1**
	[**1,1**]
chronic_yes	**1**
	[**1,1**]
businsur_yes	**1**
	[**1,1**]

```
urban                                1
                                 [1,1]

age                                  1
                                 [1,1]
--------------------------------------------
5
edu_junior                      2.274*
                         [1.030,5.017]

edu_high                        5.689**
                         [2.521,12.84]

edu_col                         8.448**
                         [3.570,19.99]

chronic_yes                     4.852**
                         [2.687,8.763]

businsur_yes                    2.540**
                         [1.514,4.264]

urban                           0.819
                         [0.501,1.339]

age                             0.950**
                         [0.925,0.976]
--------------------------------------------
N                                1391
--------------------------------------------
Exponentiated coefficients; 95% confidence intervals in brackets
* p<0.05, ** p<0.01

250 . di as result "===============================================================================
    ================================================================================

251 . di as result "Interpretation: The direction and significance of all core associations"
    Interpretation: The direction and significance of all core associations

252 . di as result "remain consistent across all three model specifications, confirming that"
    remain consistent across all three model specifications, confirming that

253 . di as result "the main findings are robust to alternative reference categories and weighting.
    the main findings are robust to alternative reference categories and weighting.

254 . di as result "===============================================================================
    ================================================================================

255 .
256 . * ==============================================================================
257 . * End of Supplementary Robustness Checks
258 . * ==============================================================================
259 .
260 .
261 . * ==============================================================================
262 . * 9. Save Stata dataset for future use
263 . * Purpose: Export the final dataset with all variables for reproducibility
264 . * ==============================================================================
265 . save "LPA_Analysis_Dataset.dta", replace
    file LPA_Analysis_Dataset.dta saved

266 .
267 . * ==============================================================================
268 . * End of do-file
269 . * ==============================================================================
270 .
    end of do-file
```
