# Supplementary material for: Latent profile analysis of eHealth literacy and its sociodemographic correlates: a cross-sectional study
Source: Front Public Health. 2026 Jul 27;14:1904244. doi: 10.3389/fpubh.2026.1904244 (PMC13454114; doi:10.3389/fpubh.2026.1904244)
Supplement: Supplementary file 5 [file Table_1.DOCX]

**Supplementary Table s1 Internal Consistency Reliability of the Chinese Version of the eHEALS**

| Subscale | Number of items | Cronbach’s α | McDonald’s ω | Reliability evaluation |
| --- | --- | --- | --- | --- |
| Total scale (item1–item8) | 8 | 0.9507 | 0.9509 | Excellent |
| Application ability (item1–item5) | 5 | 0.9425 | 0.9433 | Excellent |
| Critical evaluation ability (item6–item7) | 2 | 0.8522 | — | Good (ω unavailable for <3 items) |
| Decision-making ability (item8) | 1 | — | — | Not applicable |

Note: McDonald’s ω is only computable for subscales with at least three items. Coefficients above 0.9 indicate excellent internal consistency; values between 0.8–0.9 indicate good reliability.

**Supplementary Table S2 Exploratory Factor Analysis (8 items): Factor Loadings & Uniqueness**

| Item | Standardized Factor Loading | Uniqueness |
| --- | --- | --- |
| item1 | 0.8160 | 0.3341 |
| item2 | 0.8776 | 0.2299 |
| item3 | 0.8964 | 0.1964 |
| item4 | 0.9066 | 0.1780 |
| item5 | 0.8953 | 0.1984 |
| item6 | 0.8655 | 0.2508 |
| item7 | 0.8211 | 0.3257 |
| item8 | 0.8288 | 0.3131 |

Note: Principal component factor analysis, N=1391. Only one factor with eigenvalue >1 was extracted, explaining 74.67% of total variance. All loadings >0.6 indicate satisfactory convergent validity.

**Supplementary Table S3 EFA Eigenvalue Output (8 items)**

| Factor | Eigenvalue | Variance explained (%) | Cumulative variance (%) |
| --- | --- | --- | --- |
| Factor 1 | 5.9736 | 74.67 | 74.67 |
| Factor 2 | 0.6406 | 8.01 | 82.68 |
| Factor 3 | 0.3804 | 4.75 | 87.43 |
| Factor 4 | 0.2526 | 3.16 | 90.59 |
| Factor 5 | 0.2377 | 2.97 | 93.56 |
| Factor 6 | 0.2004 | 2.50 | 96.07 |
| Factor 7 | 0.1815 | 2.27 | 98.34 |
| Factor 8 | 0.1332 | 1.66 | 100.00 |

**Supplementary Table S4 Model Fit Indices of Unidimensional and Two-Factor Measurement Models**

| **Fit Index** | **Unidimensional 8-item Model** | **Two-factor 7-item Model** | **Acceptable Standard** |
| --- | --- | --- | --- |
| χ2(df) | 854.24 (20) | 253.25 (13) | Smaller value indicates better fit |
| P of χ2 | <0.001 | <0.001 | Non-significant preferred; significant is common in large samples |
| CFI | 0.923 | 0.974 | ≥0.90 acceptable, ≥0.95 excellent |
| TLI | 0.892 | 0.958 | ≥0.90 acceptable |
| RMSEA (90% CI) | 0.173 (0.163, 0.183) | 0.115 (0.103, 0.128) | ≤0.08 acceptable |
| SRMR | 0.043 | 0.024 | ≤0.10 excellent |
| AIC | 22086.495 | 18892.376 | Smaller = better |
| BIC | 22212.202 | 19007.607 | Smaller = better |
| CD | 0.958 | 0.983 | Closer to 1 = better explanatory power |

Note: The two-factor model excludes item 8 (single-item decision-making dimension).

**Supplementary Table S5. Fit Indices of Latent Profile Analysis and Sensitivity Analyses**

| Index | Primary Analysis  (3 dimensions) | Sensitivity Analysis 1  (8 raw eHEALS items) | Sensitivity Analysis 2 (Statistical Optimal, 2 dimensions, 6-class) | Sensitivity Analysis 2 (Theoretically Optimal, 2 dimensions, 5-class) |
| --- | --- | --- | --- | --- |
| Number of Profiles | 5 | 5 | 6 | 5 |
| AIC | 7642 | 20648 | 5801 | 6111 |
| BIC | 7757 | 20921 | 5901 | 6195 |
| Entropy | 0.952 | 0.988 | 0.922 | 0.734 |
| BLRT p-value | <0.05 | 0.0099 | 0.0099 | 1.000 |
| Minimum Subgroup Proportion | 9.85% | 8.34% | 1.58% | 5.32% |
| **Average Posterior Probability per Profile (Diagonal Values)** | 0.538, 0.882, 0.902, 0.909, 0.952 | 0.843, 0.981, 0.613, 0.973, 0.773 | **0.903, 0.924, 0.731, 0.856, 0.584, 0.776** | 0.986, 0.936, 0.622, 0.843, 0.620 |
| **Range of Diagonal Probabilities** | 0.538 – 0.952 | 0.613 – 0.981 | **0.584 – 0.924** | 0.620 – 0.986 |
| Stability across Multiple Random Seeds | Stable 5-class solution | Stable 5-class solution across 3 random seeds | Stable 6-class solution across 3 random seeds | Stable 5-class |

**Notes:** AIC = Akaike Information Criterion; BIC = Bayesian Information Criterion; BLRT = Bootstrap Likelihood Ratio Test; LPA = Latent Profile Analysis. The 5‑class solution was selected as the optimal model based on comprehensive evaluation of statistical fit, classification reliability, subgroup adequacy, and theoretical interpretability. An average posterior probability >0.70 generally indicates reliable latent subgroup assignment. In the primary analysis, Class 1 exhibited a diagonal probability of 0.538, which is below the conventional threshold but remains acceptable given the overall high entropy (0.952), adequate class size (n=157, 11.29%), and the boundary position of this class in the latent space. The other four classes all exceed 0.88, confirming excellent classification certainty overall. For the 6‑class statistical optimal solution in Sensitivity Analysis 2, Class 5 showed a diagonal probability of 0.584; however, the overall entropy remains high (0.922), and the minimum subgroup proportion is small (1.58%), suggesting that the 6‑class solution, while statistically optimal, may suffer from over‑fitting and reduced classification certainty for smaller classes. This further supports the selection of the theoretically interpretable 5‑class solution for the main analysis.

**Supplementary Table S6. Average Posterior Probability Matrices for Primary and Sensitivity Analyses**

**(a) Primary Analysis (3 dimensions, 5-class)**

| **Assigned Class** | **Profile 1** | **Profile 2** | **Profile 3** | **Profile 4** | **Profile 5** | **Average Maximum Posterior Probability** | **Sample Size** |
| --- | --- | --- | --- | --- | --- | --- | --- |
| **1** | **0.538** | 0.000 | 0.027 | 0.436 | 0.027 | 0.752 | 157 |
| **2** | 0.000 | **0.882** | 0.078 | 0.001 | 0.078 | 0.909 | 413 |
| **3** | 0.000 | 0.038 | **0.902** | 0.035 | 0.902 | 0.948 | 430 |
| **4** | 0.011 | 0.000 | 0.080 | **0.909** | 0.080 | 0.944 | 254 |
| **5** | 0.000 | 0.002 | 0.952 | 0.045 | **0.952** | 0.978 | 137 |

**(b) Sensitivity Analysis 1 (8 raw eHEALS items, 5-class)**

| **Assigned Class** | **Profile 1** | **Profile 2** | **Profile 3** | **Profile 4** | **Profile 5** | **Average Maximum Posterior Probability** | **Sample Size** |
| --- | --- | --- | --- | --- | --- | --- | --- |
| **1** | **0.843** | 0.048 | 0.002 | 0.843 | 0.092 | 0.924 | 534 |
| **2** | 0.019 | **0.981** | 0.000 | 0.019 | 0.000 | 0.995 | 347 |
| **3** | 0.007 | 0.000 | **0.613** | 0.007 | 0.265 | 0.856 | 127 |
| **4** | 0.973 | 0.027 | 0.000 | **0.973** | 0.001 | 0.995 | 116 |
| **5** | 0.088 | 0.001 | 0.040 | 0.088 | **0.773** | 0.892 | 267 |

**(c) Sensitivity Analysis 2 (Excluding decision dimension, 5‑class theoretical optimal)**

| **Assigned Class** | **Profile 1** | **Profile 2** | **Profile 3** | **Profile 4** | **Profile 5** | **Average Maximum Posterior Probability** | **Sample Size** |
| --- | --- | --- | --- | --- | --- | --- | --- |
| **1** | **0.986** | 0.000 | 0.000 | 0.014 | 0.000 | 0.997 | 74 |
| **2** | 0.000 | **0.936** | 0.025 | 0.000 | 0.038 | 0.965 | 403 |
| **3** | 0.000 | 0.238 | **0.622** | 0.001 | 0.140 | 0.808 | 22 |
| **4** | 0.039 | 0.000 | 0.056 | **0.843** | 0.062 | 0.919 | 326 |
| **5** | 0.000 | 0.016 | 0.322 | 0.041 | **0.620** | 0.801 | 566 |

**(d) Sensitivity Analysis 2 (Excluding decision dimension, 6‑class statistical optimal)**

| **Assigned Class** | **Profile 1** | **Profile 2** | **Profile 3** | **Profile 4** | **Profile 5** | **Profile 6** | **Average Maximum Posterior Probability** |
| --- | --- | --- | --- | --- | --- | --- | --- |
| **1** | **0.903** | 0.000 | 0.000 | 0.095 | 0.000 | 0.000 | 0.950 |
| **2** | 0.000 | **0.924** | 0.000 | 0.000 | 0.075 | 0.000 | 0.960 |
| **3** | 0.000 | 0.000 | **0.731** | 0.268 | 0.000 | 0.000 | 0.823 |
| **4** | 0.000 | 0.000 | 0.144 | **0.856** | 0.000 | 0.000 | 0.901 |
| **5** | 0.000 | 0.000 | 0.000 | 0.416 | **0.584** | 0.000 | 0.708 |
| **6** | 0.000 | 0.224 | 0.000 | 0.000 | 0.000 | **0.776** | 0.841 |

**Notes:** Values represent the average posterior probability of participants from each assigned latent class belonging to each latent profile. **Bolded diagonal values** indicate the average probability of correct within‑class assignment; off‑diagonal values indicate the average probability of cross‑class misassignment. An average posterior probability >0.70 generally indicates reliable classification. The 6‑class solution (panel d) is shown for completeness but was **not** selected as the final model due to a very small subgroup (1.58%) and lower interpretability. Sample sizes for panel d are omitted because this model was not retained; the last column reports the mean of each participant’s maximum posterior probability within their assigned class.

**Supplementary Table S7. Sensitivity analyses for correlates of latent eHealth literacy profiles (Class 5 vs Class 1)**

| **Covariates** | **Primary Model**  **(OR, 95% CI)** | **Age Categorical**  **(OR, 95% CI)** | **Excluding Employment**  **(OR, 95% CI)** | **Excluding Retired**  **(OR, 95% CI)** |
| --- | --- | --- | --- | --- |
| **Education** *(ref: Primary)* |  |  |  |  |
| Junior high school | **3.774 (1.506, 9.452)** | **3.349 (1.334, 8.411)** | **3.718 (1.484, 9.314)** | **3.293 (1.282, 8.461)** |
| High school | **3.895 (1.687, 8.996)** | **3.426 (1.433, 8.190)** | **3.774 (1.635, 8.711)** | **3.535 (1.463, 8.543)** |
| College and above | **4.552 (1.880, 11.023)** | **4.199 (1.713, 10.292)** | **4.502 (1.868, 10.850)** | **4.355 (1.695, 11.191)** |
| **Income** *(ref: ≥200,000)* |  |  |  |  |
| <50,000 CNY | **3.107 (1.475, 6.542)** | **3.080 (1.451, 6.537)** | **3.189 (1.507, 6.749)** | **2.787 (1.279, 6.075)** |
| 50,000–99,999 CNY | **3.090 (1.122, 8.506)** | **3.108 (1.121, 8.619)** | **3.160 (1.148, 8.699)** | **3.191 (1.107, 9.202)** |
| 100,000–149,999 CNY | **4.995 (2.126, 11.742)** | **4.808 (2.039, 11.338)** | **5.180 (2.201, 12.194)** | **4.638 (1.899, 11.328)** |
| 150,000–199,999 CNY | 1.574 (0.836, 2.961) | 1.579 (0.834, 2.987) | 1.599 (0.849, 3.011) | 1.562 (0.809, 3.017) |
| **Chronic disease: Yes** | **3.408 (1.920, 6.050)** | **3.377 (1.899, 6.007)** | **3.330 (1.884, 5.887)** | **4.057 (2.175, 7.569)** |
| **Commercial insurance: Yes** | **2.060 (1.187, 3.576)** | **1.941 (1.107, 3.405)** | **2.080 (1.197, 3.614)** | **2.558 (1.403, 4.665)** |
| **Urban residence** | **0.579 (0.340, 0.986)** | **0.586 (0.342, 1.003)** | 0.594 (0.348, 1.015) | **0.544 (0.310, 0.954)** |
| **Non-employed** | 0.740 (0.416, 1.315) | 0.763 (0.423, 1.375) | — | 1.088 (0.585, 2.025) |
| **Age (continuous)** | **0.936 (0.910, 0.962)** | — | **0.936 (0.912, 0.960)** | **0.944 (0.916, 0.972)** |

**Notes:** Only Class 5 (high eHealth literacy) vs Class 1 (low literacy) is shown for brevity; full results for all classes are available upon request. All models use the same reference categories as the primary model and are weighted by maximum posterior probability. The age‑categorical model replaces continuous age with age groups (18–34, 35–49, 50–64, ≥65). Bold indicates P < 0.05

**Supplementary Table S8. Age suppression effect: crude vs fully adjusted model**

| **Comparison** | **Crude Model (OR, 95% CI)** | **Fully Adjusted Model (OR, 95% CI)** |
| --- | --- | --- |
| **Class 2 vs Class 1** | **0.970 (0.956, 0.985)** | **0.977 (0.958, 0.997)** |
| **Class 3 vs Class 1** | **0.956 (0.942, 0.971)** | **0.952 (0.933, 0.972)** |
| **Class 4 vs Class 1** | **0.972 (0.955, 0.989)** | 0.985 (0.963, 1.008) |
| **Class 5 vs Class 1** | 1.003 (0.987, 1.019) | **0.936 (0.910, 0.962)** |

**Notes:** Both models use Class 1 (Low eHealth Literacy) as reference and are weighted by maximum posterior probability. The crude model includes age only; the fully adjusted model includes all covariates from the primary analysis (gender, education, income, chronic disease, commercial insurance, urban residence, employment status). The attenuation of the age‑Class 4 association and the emergence of a significant age‑Class 5 association after adjustment indicates partial suppression by other covariates (particularly education and income). Bold indicates P < 0.05.

**Supplementary Table S9. Robustness Checks: Alternative Reference Category and Unweighted Models**

**Table S9a. Comparison of Weighted vs Unweighted Models (Class 5 vs Class 1)**

| **Covariate** | **Weighted Model (Primary) OR (95% CI)** | **Unweighted Model OR (95% CI)** | **Difference (%)** |
| --- | --- | --- | --- |
| Education *(ref: Primary)* |  |  |  |
| Junior high school | 3.77 (1.51, 9.45) | 3.49 (1.31, 9.28) | 7.4% |
| High school | 3.90 (1.69, 9.00) | 3.51 (1.41, 8.75) | 10.0% |
| College and above | 4.55 (1.88, 11.02) | 4.16 (1.58, 10.97) | 8.6% |
| Income *(ref: ≥200,000 CNY)* |  |  |  |
| <50,000 CNY | 3.11 (1.48, 6.54) | 3.18 (1.52, 6.66) | 2.3% |
| 50,000–99,999 CNY | 3.09 (1.12, 8.51) | 3.27 (1.21, 8.79) | 5.8% |
| 100,000–149,999 CNY | 5.00 (2.13, 11.74) | 5.02 (2.17, 11.64) | 0.4% |
| 150,000–199,999 CNY | 1.57 (0.84, 2.96) | 1.64 (0.87, 3.10) | 4.5% |
| Chronic disease: Yes | 3.41 (1.92, 6.05) | 3.36 (1.84, 6.13) | 1.5% |
| Commercial insurance: Yes | 2.06 (1.19, 3.58) | 2.04 (1.17, 3.54) | 1.0% |
| Urban residence | 0.58 (0.34, 0.99) | 0.58 (0.34, 1.00) | 0.0% |
| Non-employed | 0.74 (0.42, 1.32) | 0.79 (0.44, 1.40) | 6.8% |
| Age | 0.94 (0.91, 0.96) | 0.94 (0.91, 0.97) | 0.0% |

**Notes:** Both models use Class 1 (Low eHealth Literacy) as the reference category. The weighted model uses posterior probability weights (Max_Posterior); the unweighted model does not. All OR differences between weighted and unweighted models are less than 10%, indicating that weighting does not materially affect the results. Bold indicates p < 0.05.

**Table S9b. Comparison of Class 1 Reference vs Class 4 Reference (Weighted Models)**

| **Covariate** | **Class 1 Reference (Primary) OR** | **Class 4 Reference (Moderate‑Low Balanced)** |
| --- | --- | --- |
| Comparison: Class 5 vs Reference |  |  |
| Education *(ref: Primary)* |  |  |
| Junior high school | 3.77 (1.51, 9.45) | 2.27 (1.03, 5.02) |
| High school | 3.90 (1.69, 9.00) | 5.69 (2.52, 12.84) |
| College and above | 4.55 (1.88, 11.02) | 8.45 (3.57, 19.99) |
| Chronic disease: Yes | 3.41 (1.92, 6.05) | 4.85 (2.69, 8.76) |
| Commercial insurance: Yes | 2.06 (1.19, 3.58) | 2.54 (1.51, 4.26) |
| Urban residence | 0.58 (0.34, 0.99) | 0.82 (0.50, 1.34) |
| Age | 0.94 (0.91, 0.96) | 0.95 (0.93, 0.98) |
| Comparison: Class 1 vs Class 4 |  |  |
| Education *(ref: Primary)* |  |  |
| Junior high school | 0.60 (0.26, 1.38) | — |
| High school | 1.46 (0.66, 3.21) | — |
| College and above | 1.86 (0.81, 4.25) | — |
| Chronic disease: Yes | 1.42 (0.79, 2.56) | — |
| Commercial insurance: Yes | 1.23 (0.70, 2.17) | — |
| Urban residence | 1.41 (0.90, 2.23) | — |
| Age | 1.02 (0.99, 1.04) | — |

**Notes:** Both models are weighted by posterior probability. The left column uses Class 1 (Low eHealth Literacy) as the reference category. The right column uses Class 4 (Moderate‑Low Balanced, diagonal posterior = 0.909) as the reference category. The direction and statistical significance of all core associations (education, chronic disease, age) remain consistent across both specifications. Urban residence is not significant when Class 4 is used as the reference, which is consistent with its marginal status in the primary model (p = 0.054 for Class 2, p = 0.044 for Class 5). Bold indicates p < 0.05.
